# Construction and validation of a signature for T cell-positive regulators related to tumor microenvironment and heterogeneity of gastric cancer

**DOI:** 10.3389/fimmu.2023.1125203

**Published:** 2023-08-30

**Authors:** Yangyang Guo, Yingjue Zhang, Kenan Cen, Ying Dai, Yifeng Mai, Kai Hong

**Affiliations:** ^1^ Department of Colorectal Surgery, The First Affiliated Hospital of Ningbo University, Ningbo, Zhejiang, China; ^2^ Department of Molecular Pathology, Division of Health Sciences, Graduate School of Medicine, Osaka University, Suita, Osaka, Japan; ^3^ Medicine School, Ningbo University, Ningbo, Zhejiang, China

**Keywords:** positive regulator, T cell, gastric cancer, prognosis, tumor microenvironment

## Abstract

**Background:**

Positive regulators of T cell function play a vital role in the proliferation and differentiation of T cells. However, their functions in gastric cancer have not been explored so far.

**Methods:**

The TCGA-STAD dataset was utilized to perform consensus clustering in order to identify subtypes related to T cell-positive regulators. The prognostic differentially expressed genes of these subtypes were identified using the least absolute shrinkage and selection operator (LASSO) regression analysis. To validate the robustness of the identified signature, verification analyses were conducted across the TCGA-train, TCGA-test, and GEO datasets. Additionally, a nomogram was constructed to enhance the clinical efficacy of this predictive tool. Transwell migration, colony formation, and T cell co-culture assays were used to confirm the function of the signature gene in gastric cancer and its influence on T cell activation.

**Results:**

Two distinct clusters of gastric cancer, related to T cell-positive regulation, were discovered through the analysis of gene expression. These clusters exhibited notable disparities in terms of survival rates (P = 0.028), immune cell infiltration (P< 0.05), and response to immunotherapy (P< 0.05). Furthermore, a 14-gene signature was developed to classify gastric cancer into low- and high-risk groups, revealing significant differences in survival rates, tumor microenvironment, tumor mutation burden, and drug sensitivity (P< 0.05). Lastly, a comprehensive nomogram model was constructed, incorporating risk factors and various clinical characteristics, to provide an optimal predictive tool. Additionally, an assessment was conducted on the purported molecular functionalities of low- and high-risk gastric cancers. Suppression of DNAAF3 has been observed to diminish the migratory and proliferative capabilities of gastric cancer, as well as attenuate the activation of T cells induced by gastric cancer within the tumor microenvironment.

**Conclusion:**

We identified an ideal prognostic signature based on the positive regulators of T cell function in this study.

## Introduction

Gastric cancer, a highly malignant tumor of the digestive system, is often detected at advanced stages, resulting in a poor prognosis for numerous patients (1). Despite the potential for early detection through gastroscopy, the considerable heterogeneity of gastric cancer poses a significant obstacle to this approach. In 2018, gastric cancer ranked as the third leading cause of cancer-related deaths globally, claiming approximately 784,000 lives (2). Furthermore, it stands as the fifth most frequently diagnosed malignant tumor, with over 1 million new cases reported each year (2). A deeper understanding of gastric cancer, regardless of genetic, molecular, or phenotypic levels, helps in managing this disease better and reduces the financial burden on patients.

T cells, including CD4+ and CD8+ T cells, play vital roles in the human immune system (3). CD4+ T cells are helper T cells that receive signals delivered by macrophages after phagocytosis, aiding in the creation of antibodies (4). In addition, these cells promote the effector and memory functions of CD8+ cytotoxic T lymphocytes (CTLs) and assist in overcoming the negative regulation of CTLs (5). Accumulating evidence confirms that CD4+ T cells target tumor cells by directly eliminating them *via* cytolysis or by indirectly regulating the tumor microenvironment (TME) (6, 7). In secondary lymphoid organs, CDT4+ T cells augment the quality and magnitude of CTL and B-cell responses (8, 9). In the second T cell-priming step, CD4+ and CD8+ T cells recognize antigens specific to them on the same dendritic cell (DC), aiding the DC in optimizing antigen presentation and deliver signals to CD8+ T cells for promoting their colonies and differentiation (8–13). These findings demonstrate the importance of T cells in immunity and antitumor functions. Certain regulators regulate the proliferation and function of T-cells. The CRISPR-based loss-of-function screens have been used to identify several negative regulators (14–16). Recently, Legut et al. used a genome-scale open reading frame (ORF) screen to find 33 positive regulators of T cells that positively modulated T cell functions (17). For instance, although the lymphotoxin-β receptor (*LTBR*) has low expression in T cells, when over-expressed, it induces transcriptional and epigenomic remodeling, promoting the effector functions of T cells and helps resist the exhaustion of exposure to chronic stimulations (17). It was demonstrated that *LTBR* and other positive regulators increased antigen-specific responses of chimeric antigen receptor T cells and γδ T cells (17). Therefore, T cell-positive regulators exhibit potential for anti-tumor therapy. However, prior studies have not investigated the function and expression of T cell regulators in tumor cells, or their correlation with the TME, containing diverse tumor-related T cells.

Tumor cells originate from human cells and can easily escape elimination by the immune system. TME is a unique environment containing tumor, immune, and stromal cells and a matrix related to tumor progression and metastasis (18). The composition of resident cells in the TME, including CTLs, CD4+ helper T cells, DCs, and macrophages, is different from the human immune system, revealing the specific characteristics of the TME (19–22). Many studies suggest that the context of the TME reflects immunotherapy responses and chemotherapy benefits and is also associated with the prognosis of patients (23–27). Zeng et al., identified three TME-related phenotypes of gastric cancer with TME infiltration patterns, which were demonstrated to be related to genomic characteristics and clinicopathological features. TME was revealed to be substantially relevant to gastric cancer, both at the genomic and clinical levels (28). Thus, the TME in gastric cancer was comprehensively analyzed in our study. Jiang et al. discovered Tumor immune dysfunction and exclusion (TIDE) as a model that represents two key mechanisms of tumor immune evasion: the impairment of T cell function in tumors with a high presence of cytotoxic T lymphocytes (CTL) and the hindrance of T cell infiltration in tumors with low CTL levels. TIDE has the potential to effectively predict the prognosis of patients undergoing anti-PD1 or anti-CTLA4 treatment, surpassing other biomarkers like PD-L1 levels and mutation load (29).

In this study, two T cell positive regulator-based subtypes with different immune cell infiltration patterns and immunotherapy responses were identified. A stable signature was confirmed with prognostic T cell-positive regulator-related genes, showing a significant correlation with TME, TMB, clinical characteristics, immunotherapy responses, and diverse drug sensitivities. Moreover, this signature was a potential independent predictive factor, and the nomogram constructed with the signature precisely predicted the survival of each patient with gastric cancer.

## Results

### Exploration of differentially expressed genes and consensus cluster construction of T cell-positive regulator

**Figure 1:**
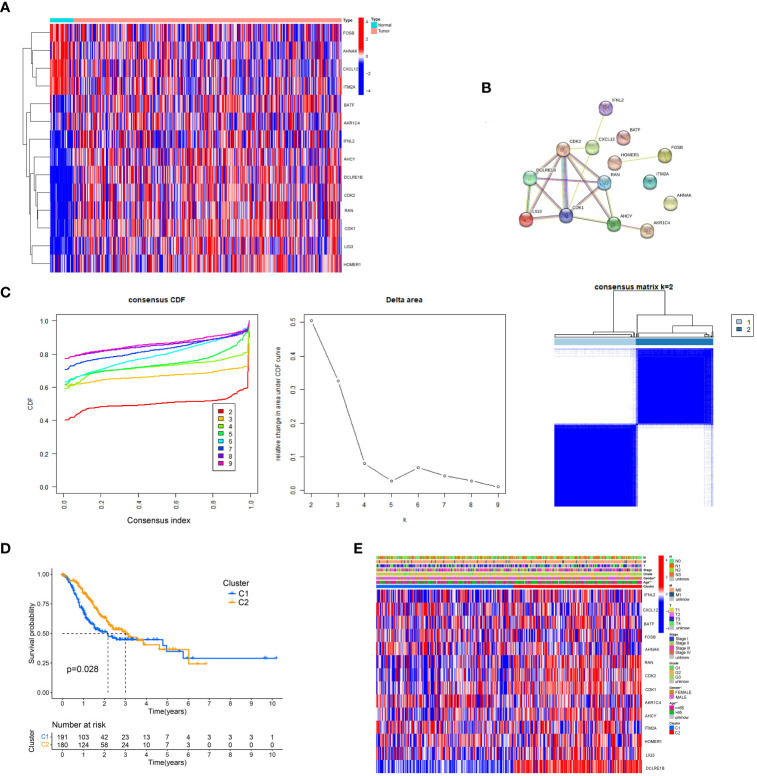
Consensus clustering of T cell-positive regulators in gastric cancer. **(A)** A heatmap of expression of T cell-positive regulator DEGs in gastric cancer and normal tissues. **(B)** A PPI network of T cell-positive regulator DEGs. **(C)** Consistent clustering of T cell-positive regulators. **(D)** Survival analysis of T cell-positive regulator cluster 1 and 2. **(E)** Heatmap of expression of T cell-positive regulators in cluster 1 and 2.

To explore whether the 33 T cell-positive regulators were abnormally expressed in gastric cancer (17) (**Supplementary File S1**), we analyzed the mRNA expression data and clinical information across TCGA-STAD and found 14 T cell-positive regulator differentially expressed genes (DEGs) from tumor versus normal tissue. Results revealed that T cell-positive regulators might function in gastric cancer progression (**Figure 1A**). Additionally, correlations between the DEGs were analyzed using the STRING online tool (https://string-db.org/), indicating the interaction of T-cell positive regulator DEGs in humans (**Figure 1B**). To acquire gastric cancer subtypes related to T cell-positive regulators, we conducted consistency clustering with 33 T cell-positive regulators; the clustering had the best stability (**Figure 1C**) when k = 2. The clarity of the edge of each cluster in the heatmap is notable, and the smoothness of the k=2 trend within the u=0.1-0.9 range (X-Axis) in the CDF plot is evident. To reveal the superiority of clustering, we used a K-M analysis presenting the prognostic capacity showing the poor outcome of cluster 1 across TCGA-STAD (P = 0.028) (**Figure 1D**). Moreover, the heatmap showed a good separation of expression of T cell-positive regulator DEGs between the two clusters, and a significant correlation between clustering, age, and gender (P< 0.05) (**Figure 1E**).

### TME and immunotherapy response in T cell-positive regulator-related subtypes

**Figure 2:**
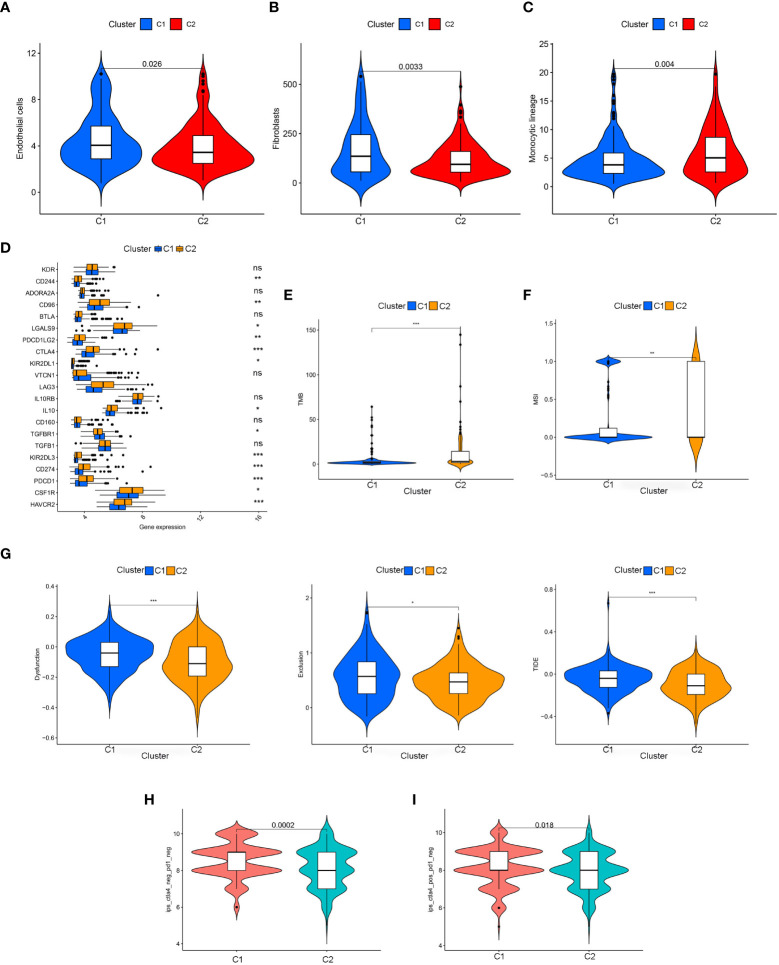
Immune cell infiltration analysis in cluster 1 and 2 about **(A)** endothelial cells, **(B)** fibroblasts, **(C)** monocytic lineage; Biomarkers of immunotherapy responses in cluster 1 and 2. **(D)** Expression of immune-related genes in cluster 1 and 2. **(E)** TMB score in cluster 1 and 2. **(F)** MSI score in cluster 1 and 2. **(G)** TIDE score in cluster 1 and 2. **(H)** IPS of cluster 1 and in CTLA4- and PD1- subgroup. **(I)** IPS of cluster 1 and 2 in CTLA4+ and PD1- subgroup. *P < 0.05, **P < 0.01, and ***P <0.001. ns, no statistical difference.

TME plays a vital role in tumor progression (30). To decipher the function of TME related to T cell-positive regulators in gastric cancer, we analyzed immune cell infiltration in two clusters across TCGA-STAD. This confirmed the significantly higher number of CD8+ T cells (P = 0.006), cytotoxic lymphocytes (P = 1.9e-05), monocytic lineage (P = 0.004), T cell (P = 0.0099), and NK cell (P = 0.0065) infiltrations in cluster 2 gastric cancer, along with the higher endothelial cell (P = 0.026) and fibroblast (P = 0.0033) infiltrations in cluster 1 (**Figures 2A–C**; **Supplementary Figure S1A**). Multiple lines of evidence indicated that TME was closely associated with tumor prognoses and immunotherapy response. Thus, we sequentially addressed the other focus of this study, the immunotherapy response. The expression of immune checkpoint genes was analyzed and visualized, showing multiple significantly expressed differences in immune checkpoint genes (P< 0.01) (**Figure 2D**). This indicated the ability to select potential immune checkpoint treatments for gastric cancer clusters. Tumor mutation burden (TMB) (P< 0.001) (**Figure 2E**), microsatellite instability (MSI) (P< 0.01) (**Figure 2F**), and TIDE (P< 0.001) (**Figure 2G**) were identified to be significantly associated with T cell-positive regulator-related clustering, all of which reported advantageous signatures predicting immunotherapy response. Furthermore, we found that in both CTLA4- PD1- (P = 0.0002) and CTLA4- PD1+ (P = 0.018) subgroups, cluster 1 gastric cancer patients had a higher immunophenoscore (IPS), indicating better immunotherapy response (31) (**Figures 2H, I**).

### Establishment of a T cell-positive regulator-related signature

**Figure 3:**
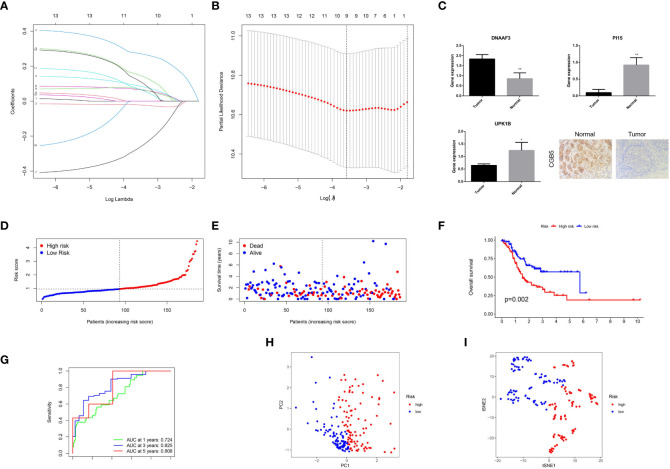
Establishment of T cell positive regulator-related score. **(A)** LASSO regression of the prognostic genes. **(B)** Cross-validation for tuning parameter selection in the LASSO regression. **(C)** Expression of signature genes in gastric tumor and normal cells. **(D)** Distribution of risk score in TCGA-train. **(E)** Survival status plot and survival time of low- and high-risk gastric cancer in TCGA-train. **(F)** Survival analysis of low- and high-risk gastric cancer in TCGA-train. **(G)** ROC curves analysis of low- and high-risk gastric cancer across TCGA-train. **(H)** PCA analyses of low- and high-risk gastric cancer in TCGA-train. **(I)** t-SNE analysis of low- and high-risk gastric cancer in TCGA-train. **P < 0.01.

**Figure 4:**
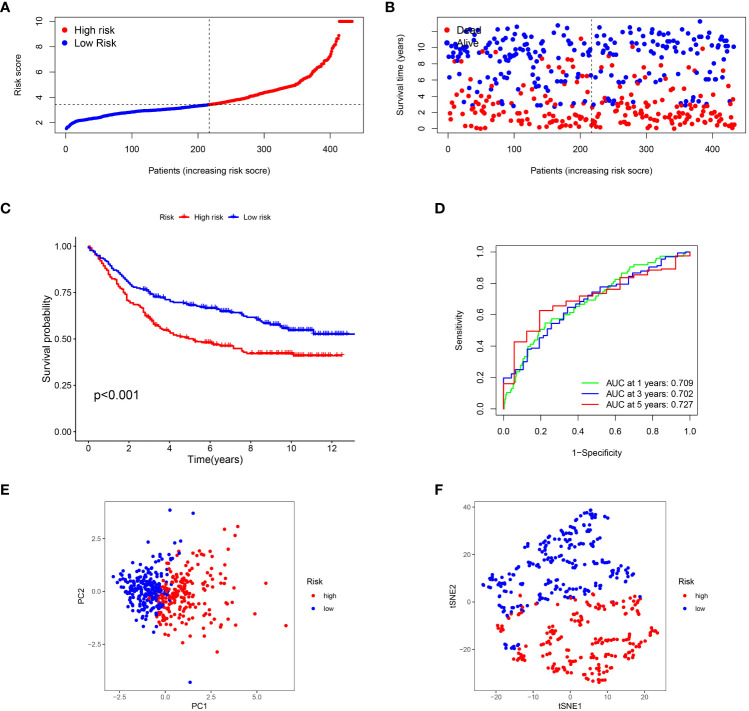
Verification of the T cell-positive regulator-related score in GEO dataset. **(A)** Distribution of risk score in GEO dataset. **(B)** Survival status plot and survival time of low- and high-risk gastric cancer in GEO dataset. **(C)** Survival analysis of low- and high-risk gastric cancer in GEO dataset. **(D)** Survival analysis of low- and high-risk gastric cancer in GEO dataset. **(E)** PCA analyses of low- and high-risk gastric cancer in GEO dataset. **(F)** t-SNE analysis of low- and high-risk gastric cancer in GEO dataset.

To obtain a better biomarker for T cell-positive regulators predicting prognoses and guiding treatments, we first calculated the prognostic DEGs between clusters 1 and 2 gastric cancer (P< 0.05) by limma algorithm and univariate Cox regression analysis across the TCGA-STAD dataset. A total of 13 prognostic DEGs were identified (**Supplementary File S2**). Then the TCGA-STAD dataset was randomly divided into TCGA-train and TCGA-test subsets by a ratio of 1:1. In TCGA-train dataset, least absolute shrinkage and selection operator (LASSO) Cox regression was performed with 13 prognostic DEGs to identify 9 optimal subsets of prognostic gene (**Figures 3A, B**). Subsequently, a multivariate Cox regression was conducted to construct a T cell-positive regulator-related signature comprising of 4 genes. The risk score was calculated with the formula: (0.344996567879663) * Expr*CGB5* + (0.341413035547099) * Expr*PI15* + (0.147288794725853) * Expr*UPK1B* + (-0.5123620001833) * Expr*DNAAF3* (**Supplementary File S3**). The results obtained from reverse transcription-quantitative polymerase chain reaction (RT-qPCR) and immunohistochemical indicated that the expression levels of CGB5, UPK1B, and PI15 were downregulated in gastric tumor cells, while DNAAF3 was upregulated (**Figure 3C**). Additionally, the investigation of the expression of four signature genes in the tumor immune microenvironment through single-cell sequencing analysis confirmed the presence of CGB5, PI15, UPK1B, and DNAAF3 in the T cell cluster (**Supplementary Figure S1B**). High-risk gastric cancer was determined by the median value of T cell-positive regulator-related score (**Figures 3D, E** **)** and showed poor prognoses according to the K-M analysis across TCGA-train (P = 0.002) (**Figure 3F**). Subsequently, to verify the predictive ability of the T cell-positive regulator-related signature, we plotted a scatter plot and conducted a receiver operating characteristic (ROC) analysis across TCGA-train dataset (**Figure 3G**). It was revealed that patients with high scores had shorter survival times and the signature had an excellent sensitivity for predicting 1-, 3-, and 5-year survival (**Figures 3D, G**). Principal component analysis (PCA) and t-distributed stochastic neighbor embedding (t-SNE) analysis with TCGA-STAD proved the effective separation of low- and high-risk gastric cancer (**Figures 3H, I**). The analyses mentioned above were conducted using the GEO dataset in combination with GSE84437 and GSE13861, as well as the TCGA-test dataset, to verify the effectiveness of the signature established in this study. All the results, as depicted in **Figure 4**, **Supplementary Figure S2**, confirmed its superior effectiveness.

### Construction of the nomogram and verifying its stability

**Figure 5:**
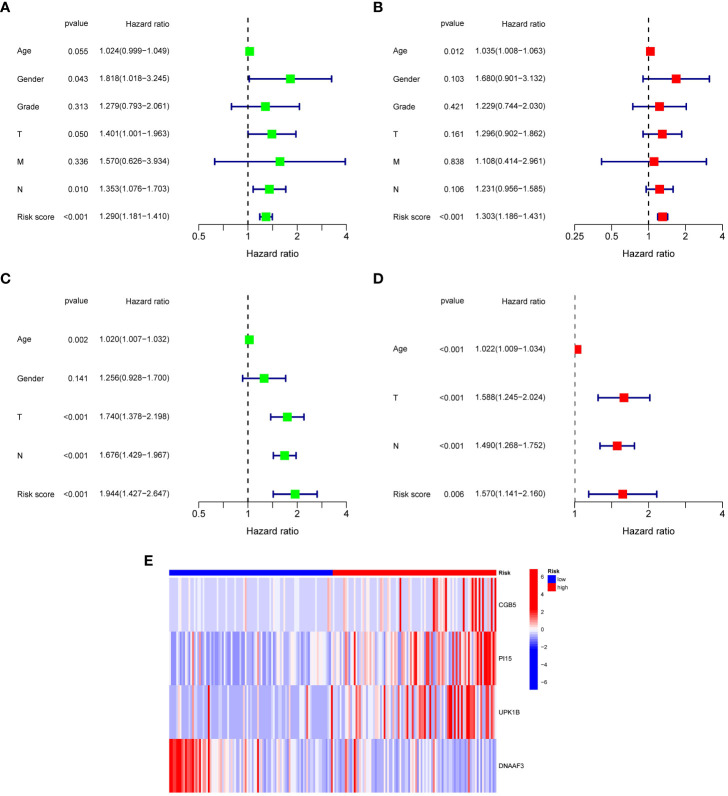
Independently prognostic ability analysis and expression of 4 genes consisting of the T cell-positive regulator-related score. **(A)** Univariate analysis of risk score and clinical characteristics in TCGA-STAD. **(B)** Multivariate analysis of risk score and clinical characteristics in TCGA-STAD. **(C)** Univariate analysis of risk score and clinical characteristics in GEO dataset. **(D)** Multivariate analysis of risk score and clinical characteristics in GEO dataset. **(E)** Heatmap pf expression of 4 genes constructing the T cell-positive regulator-related score.

**Figure 6:**
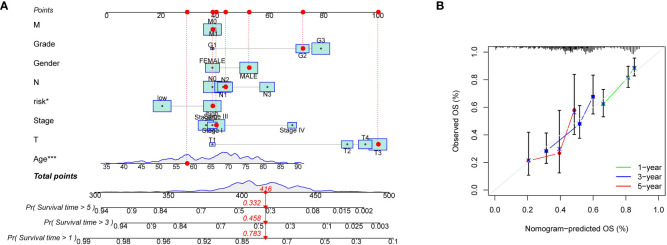
Construction of the prognostic nomogram. **(A)** Nomogram of T cell-positive regulator-related score and clinical characteristics of TCGA-STAD. **(B)** Calibration plot for evaluating the predictive ability of the nomogram at 1-, 3-, and 5-years. *P < 0.05 and ***P < 0.001.

While the signature had a stable capacity to predict survival of low- and high-risk gastric cancer, it could only divide patients into these two groups, with low-risk patients having better prognoses. However, doctors need a precise and comprehensive tool to predict the survival of each patient. To address this, we developed an intuitive visual tool called a robust nomogram. First, we performed univariate and multivariate Cox regression analyses across TCGA-STAD and GEO datasets to identify independent prognostic factors for gastric cancer. Results demonstrated that age, T stage, N stage, and the signature could act as such factors (**Figures 5A–D**). In addition, the low- and high-risk gastric cancer showed distinct expression of signature genes (**Figure 5E**). We sequentially established a nomogram with age, sex, clinical stage, tumor grade, T stage, M stage, and N stage, with which the 1-, 3-, and 5-year survival of each patient could be predicted (**Figure 6A**). The calibration curve showed a robust and independent predictive probability of the nomogram (**Figure 6B**). The findings of this study indicate that age, gender, clinical stage, tumor grade, and nomogram are all significant independent prognostic factors. The receiver operating characteristic (ROC) analysis revealed that the nomogram exhibited the highest area under the curve (AUC) value of 0.757, while the signature had a slightly lower AUC value of 0.707.

### Subgroup analysis of the signature

**Figure 7:**
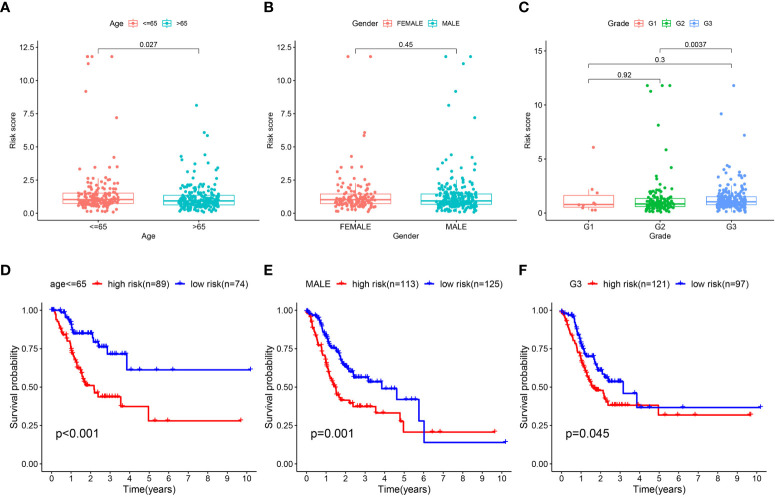
Subgroup analysis of T cell-positive regulator-related risk score. **(A–C)** Difference of T cell-positive regulator-related risk score in gastric cancer with different age, gender, and tumor grade; survival analysis of low- and high-risk gastric cancer in subgroups of **(D–F)** age ≤ 65, male, and tumor grade 3.

To explore whether gastric cancer patients with different characteristics had distinct risk scores, we analyzed the differences in risk scores of the different subgroups. Significant differences were observed in the TCGA-STAD dataset (**Figures 7A–C** and **Supplementary Figure S3A**) between patients with age > 65 and ≤ 65 years, tumor grade 2 and 3, M stage 0 and 1, N stage 0 and 2, N stage 0 and 3, N stage 1 and 2, clinical stage I and II, clinical stage I and III, clinical stage I and IV, clinical stage III and IV, T stage 1 and 2, T stage 1 and 3, T stage 1 and 4. Significant differences (P< 0.05) were observed in the GEO dataset among patients categorized into different T and N stages, including T stage 1 and 2, T stage 1 and 3, T stage 1 and 4, T stage 2 and 3, T stage 2 and 4, T stage 3 and 4, N stage 0 and 1, N stage 0 and 2, N stage 0 and 3, N stage 1 and 3, and N stage 2 and 3, as depicted in **Supplementary Figure S3B**. Moreover, we found significant differences in survival in the subgroups of age > 65 years (P< 0.001), age ≤ 65 years (P< 0.001), male sex (P = 0.001), tumor grade 3 (P = 0.045), N stage 1-3 (P = 0.014), T stage 3-4 (P< 0.001), M stage 0 (P = 0.018), M stage 1 (P = 0.034), and clinical stage III-IV (P = 0.008) (**Figures 7D–F**, **Supplementary Figure S3C**). Besides, significantly higher risk score was identified in the C1 cluster than that in the C2 cluster (**Supplementary Figure S3D**).

### Correlation between TME and the signature

**Figure 8:**
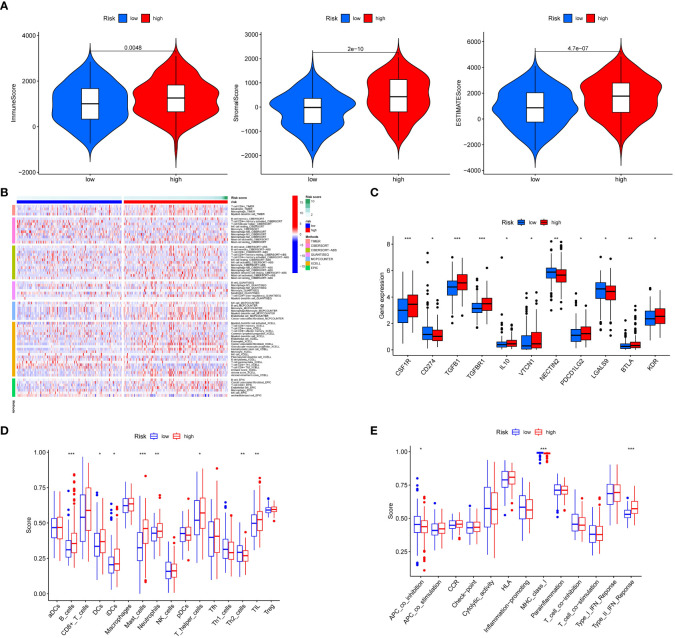
Relationship between T cell-positive regulator-related risk score and human immunity. **(A)** Immune purity of low- and high-risk gastric cancer. **(B)** Immune cell infiltrations including CD4 T cell, B cell, macrophage, neutrophil, myeloid dendritic cell, monocyte, NK cell, mast cell, endothelial cell, and cancer associated fibroblast in low- and high-risk gastric cancer by the “TIMER,” “CIBERSORT,” “CIVERSORT-ABS,” “QUANTISEQ,” “MCPCOUNTER,” “XCELL,” and “EPIC” algorithms. **(C)** Expression differences of immune-related genes in low- and high-risk gastric cancer. **(D)** Immune cell score in low- and high-risk risk gastric cancer. **(E)** Immune function score in low- and high-risk gastric cancer. *P < 0.05, **P < 0.01, and ***P <0.001.

The TME consists of tumor, immune, and stromal cells, and the extracellular matrix (ECM), which are reported to play a vital role in tumor progression (32). Previous evidence demonstrated that the composition of TME in both cells and ECM partly accounts for the distinct outcomes of patients (33). To examine the difference between the TME in low- and high-risk gastric cancers, we performed a comprehensive analysis. First, we calculated the StromalScore, ImmuneScore, and ESTIMATEScore of the two subtypes and found a significantly higher tumor purity in low-risk gastric cancer (P< 0.05) (**Figure 8A**). With the development of algorithms and a deeper understanding of immune cell infiltration, diverse algorithms for immune cell infiltration have emerged. Here, we globally analyzed the immune cell infiltrations in gastric cancer subtypes using the “TIMER,” “CIBERSORT,” “CIVERSORT-ABS,” “QUANTISEQ,” “MCPCOUNTER,” “XCELL,” and “EPIC” algorithms (**Figure 8B**). The TIMER algorithm revealed a greater presence of CD4 T cells, neutrophils, macrophages, and myeloid dendritic cells in high-risk gastric cancer. Additionally, the CIBERSORT algorithm detected higher levels of M2 macrophages, monocytes, B cell memory, and activated mast cells in high-risk gastric cancer. Furthermore, the QUANTISEQ algorithm determined an increased abundance of B cells, M2 macrophages, and CD4 T cells in high-risk gastric cancer. The MCPCOUNTER algorithm revealed that gastric cancer with a high-risk profile exhibited elevated levels of myeloid dendritic cells, endothelial cells, and cancer-associated fibroblasts. Similarly, the XCELL algorithm demonstrated that high-risk gastric cancer displayed increased quantities of myeloid dendritic cells, endothelial cells, eosinophils, cancer-associated fibroblasts, and monocytes. Additionally, the EPIC algorithm identified higher levels of B cells, cancer-associated fibroblasts, CD4 T cells, endothelial cells, and macrophages in high-risk gastric cancer. Subsequently, we conducted an assessment of the expression levels of several established immune-related genes, namely CSF1R, CD274, TGFB1, TGFBR1, IL10, VTCN1, NECTIN2, PDCD1LG2, LGALS9, BTLA, and KDR, in order to demonstrate the differential expression patterns observed between low- and high-risk gastric cancer cases (**Figure 8C**). These findings serve to reinforce the association between immune cell infiltration and the aforementioned signature. Furthermore, we conducted an estimation of the immune cell and immune function scores pertaining to the two distinct subtypes of gastric cancer. Our analysis revealed that B cells, dendritic cells (DCs), mast cells, neutrophils, T helper cells, Th2 cells, tumor-infiltrating lymphocytes (TILs), antigen-presenting cell (APC) co-inhibition, and type II interferon (IFN) response exhibit varying involvement in the invasion of low- and high-risk gastric cancer subtypes (**Figures 8D, E**). Together, these results showed that the TME was distinct in low- and high-risk gastric cancer, which might influence tumor development and prognosis.

### Immunotherapy response and drug sensitivity

**Figure 9:**
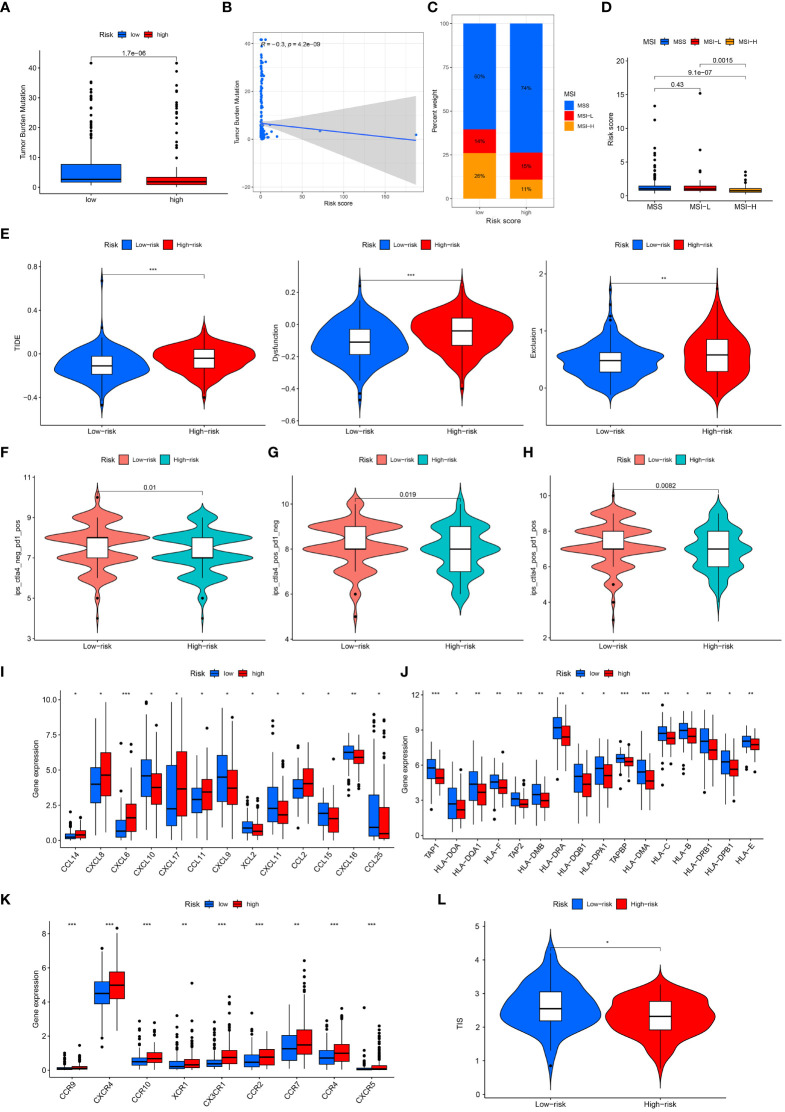
Immunotherapy response in low- and high-risk gastric cancer. **(A, B)** Correlation of T cell-positive regulator-related risk score and TMB. **(C, D)** Correlation of T cell-positive regulator-related risk score and MSI. **(E)** TIDE score in low- and high-risk gastric cancer. **(F)** IPS of low- and high-risk gastric cancer in CTLA4- PD1+ subgroup. **(G)** IPS of low- and high-risk gastric cancer in CTLA4+ PD1- subgroup. **(H)** IPS of low- and high-risk gastric cancer in CTLA4+ PD1+ subgroup. **(I–K)** The expression of immune regulatory factors between low- and high-risk groups. **(L)** The TIS score between low- and high-risk groups.

**Figure 10:**
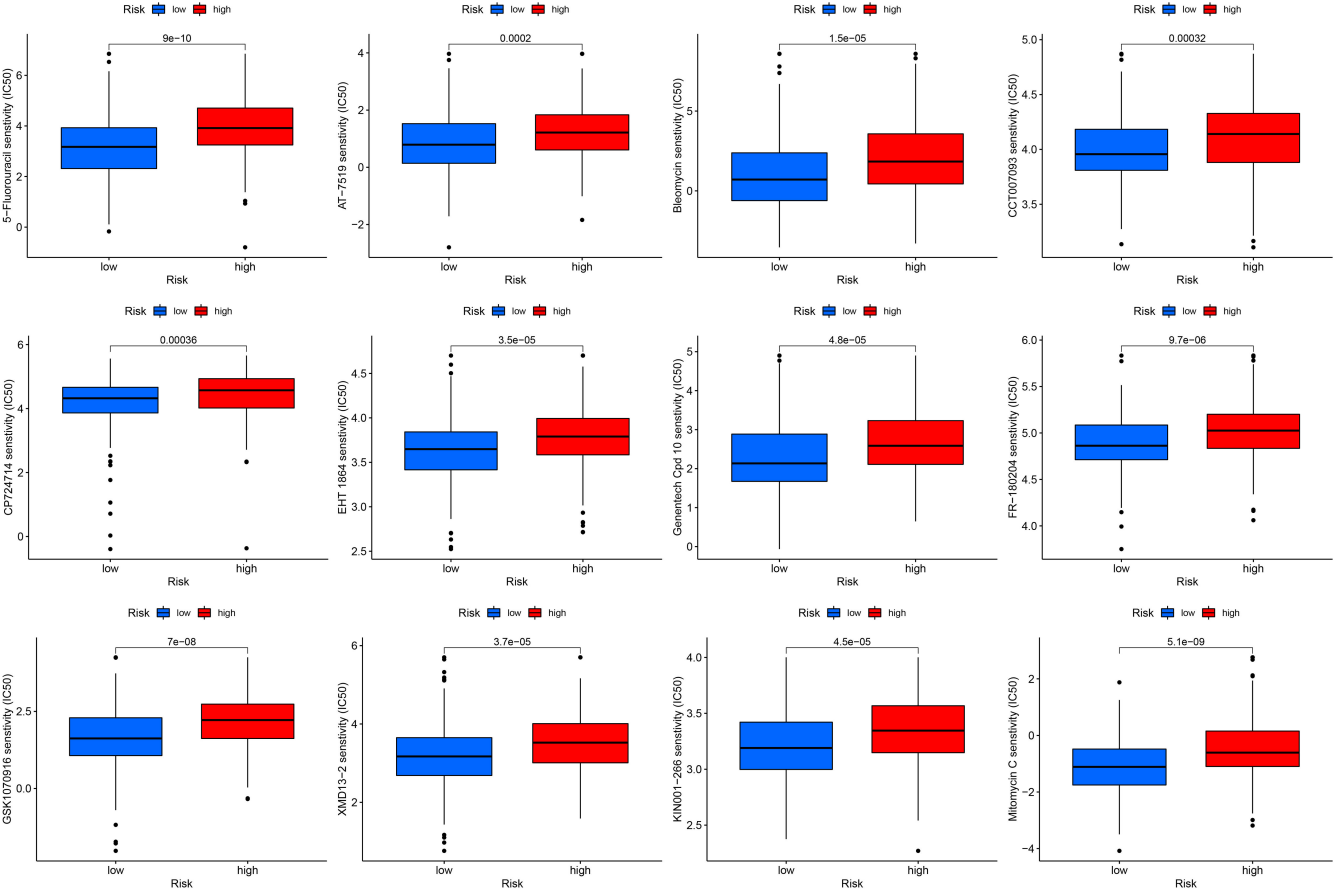
Drug sensitivity of low- and high-risk gastric cancer.

An excellent biomarker needs not only the predictive ability of survival, but also the capacity to speculate treatment response. To test whether the signature developed in this study achieved this, we calculated TMB, MSI, TIDE, and IPS to examine immunotherapy response. Low-risk gastric cancer exhibited a higher tumor mutational burden (TMB) score, and there was a negative correlation between the risk score and TMB score (**Figures 9A, B**). Additionally, the low-risk subgroup displayed a higher proportion of microsatellite instability-high (MSI-H) patients and a lower proportion of microsatellite stable (MSS) patients (**Figure 9C**). Furthermore, MSI-H gastric cancer exhibited a lower risk score compared to MSS and microsatellite instability-low (MSI-L) gastric cancer (**Figure 9D**). Notably, low-risk gastric cancer demonstrated a lower Tumor Immune Dysfunction and Exclusion (TIDE) score, suggesting that these patients may derive greater benefits from immunotherapy (**Figure 9E**). Concurrently, the CTLA4– PD1+, CTLA4+ PD1–, and CTLA4+ PD1+ subgroups exhibited a notable advantage in immunotherapy for low-risk gastric cancer (**Figures 9F, E, H**). The association between the signature and various immune regulatory factors was examined, revealing a significant disparity between our model and key immune regulatory factors such as CCL14 (P< 0.05), CXCL17 (P< 0.05), HLA-B (P< 0.05), and CCR10 (P< 0.001) (**Figures 9I–K**). Additionally, it was observed that low-risk gastric cancer exhibited a higher T cell-inflamed score (TIS) compared to high-risk gastric cancer (P< 0.05). Moreover, in the analysis of drug sensitivity, low-risk gastric cancer demonstrated enhanced responsiveness to 5-fluorouracil, AT-7519, bleomycin, CCT007093, CP724714, EHT1864, Genentech Cpd 10, FR-180204, GSK1070916, XMD13-2, and KIN001-266 (**Figure 10**). Furthermore, the cohort receiving anti-PD-L1 treatment was employed to predict the immunotherapy response in both low- and high-risk gastric cancer. The results indicated that low-risk gastric cancer may derive greater benefits from immunotherapy (**Supplementary Figure S3E**).

### Putative biological function associated T cell-positive regulator-related signature

**Figure 11:**
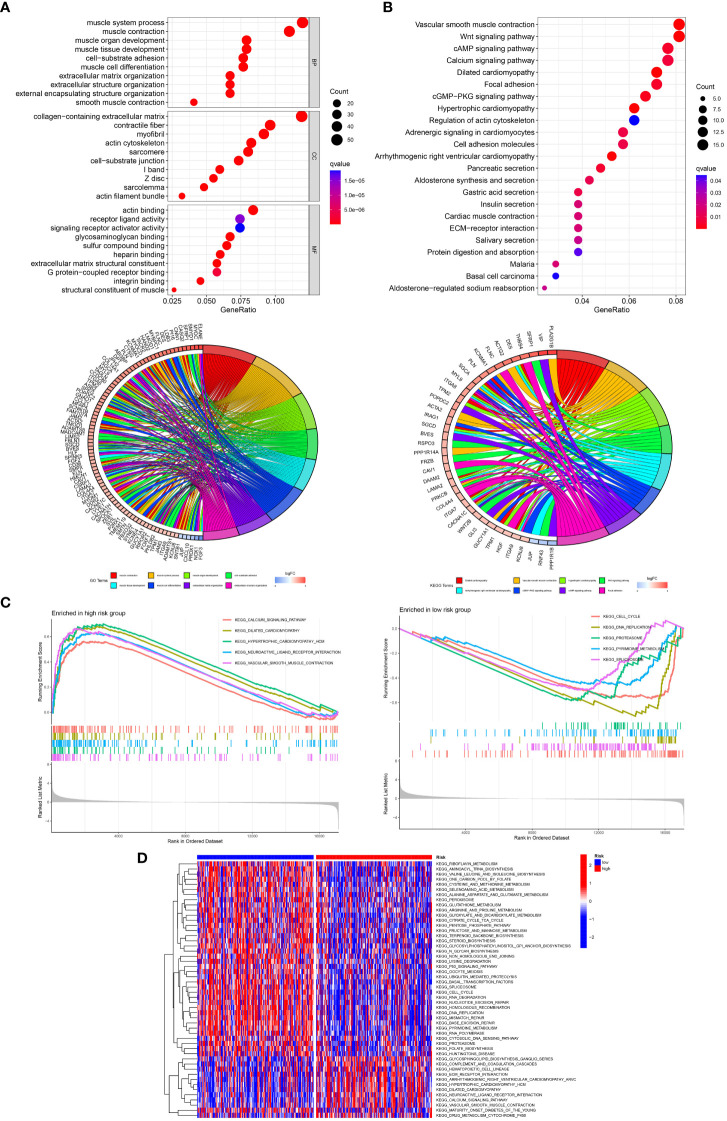
Functional enrichment analyses of DEGs between low- and high-risk gastric cancer. **(A)** GO analysis. **(B)** KEGG analysis. **(C)** GSEA. **(D)** GSVA.

To explore the biological processes related to the T cell-positive regulator, we conducted a series of functional enrichment analyses, including Gene Ontology (GO), Kyoto Encyclopedia of Genes and Genomes (KEGG), gene set enrichment (GSEA), and gene set variation (GSVA) analyses. According to the results, “muscle system process,” “muscle contraction,” and “muscle organ development” in BP analysis; “collagen-containing extracellular matrix,” “contractile fiber,” and “myofibril” in CC analysis; as well as “actin binding,” “receptor ligand activity,” and “signaling receptor activator activity” in the MF analysis were the top three functional annotations among these GO analyses (**Figure 11A**). The “vascular smooth muscle contraction,” “Wnt signaling pathway,” “cAMP signaling pathway,” “calcium signaling pathway,” “dilated cardiomyopathy,” and “focal adhesion” were the top six pathways related to T cell-positive regulation (**Figure 11B**). Furthermore, pathways like “calcium signaling pathway,” “dilated cardiomyopathy,” or “vascular smooth muscle conteraction” were highly enriched in high-risk gastric cancer, as well as “cell cycle,” “DNA replication,” or “proteasome” were more related to low-risk gastric cancer (**Figure 11C**). From the GSVA analysis, hallmarks such as “glycosphingolipid biosynthesis ganglio series” or “calcium signaling pathway” were high-regulated in high-risk subtypes, while “P53 signaling pathway,” “cell cycle,” or “pentose phosphate pathway” were highly regulated in low-risk disease (**Figure 11D**). Functional enrichment analyses provided comprehensive speculation of putative molecular actions that helped us identify T cell-positive regulators.

### Knockdown of DNAAF3 in gastric cancer cell suppressing tumor proliferation and migration and T cell viability

**Figure 12:**
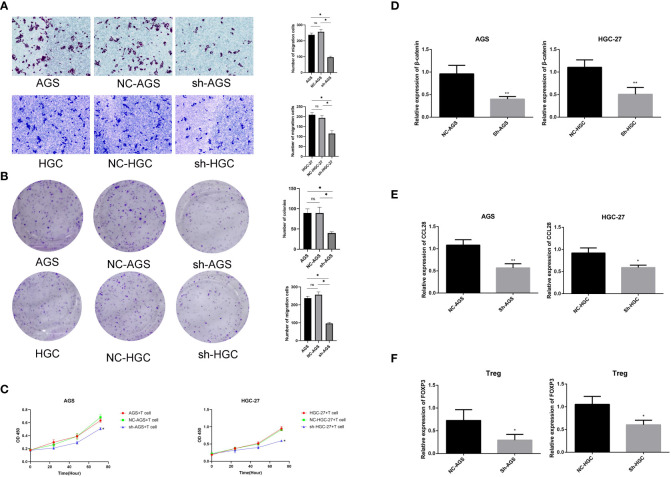
Experiments of knockdown of *DNAAF3* in gastric cancer **(A)** Transwell migration assay. **(B)** Colony formation assay. **(C)** T cell and gastric cancer cell co-culture assay. **(D)** Expression of β-catenin in gastric cancer. **(E)** Expression of CCL28 in gastric cancer. **(F)** Expression of FOXP3 in T cells. *P < 0.05 and **P < 0.01.

The Transwell migration assay demonstrated a significant decrease in the number of migrating cells following the knockdown of DNAAF3 in HGC-27 and AGS cells (P< 0.05) (**Figure 12A**). Additionally, the colony formation capacity of DNAAF3 knockdown cells was significantly diminished compared to the negative control groups (P< 0.05) (**Figure 12B**). To investigate the impact of DNAAF3 on T cells in gastric cancer, a co-culture experiment was conducted with gastric cancer cells. Results of the CCK8 assay supported that the knockdown of *DNAAF3* will decrease the ability of gastric cancer in activating T cell viability (P< 0.05) (**Figure 12C**).

In a previous publication, it was reported that the suppression of gastric cancer progression through inhibition of Treg cell infiltration can be achieved by blocking β-Catenin-induced CCL28 (34). Additionally, the KEGG enrichment analysis revealed that the Wnt/β-Catenin pathway was enriched. In order to investigate the mechanism by which DNAAF3 in gastric cancer cells affects T cells, the relevant marker was assessed using RT-qPCR. The results demonstrated that the knockdown of DNAAF3 led to a decrease in the expression of β-Catenin and its downstream target CCL28 in gastric cancer (**Figures 12D, E**), resulting in the suppression of Treg cell (FOXP3+) infiltration (**Figure 12F**).

## Discussion

Although none of the positive regulators of T cell functions have been studied in tumor cells, let alone in gastric cancer, a strong relationship between gastric cancer and immunity has been widely confirmed (35, 36). *Helicobacter pylori* are one of the most important risk factors for gastric cancer (37). Chronic atrophic gastritis caused by Helicobacter pylori leads to the malignancy of gastric epithelial cells (38). Therefore, the complex mechanisms of gastric cancer contribute to the complicated immune microenvironment, where we proposed that immune cells, such as T cells, probably function. We evaluated positive regulators in gastric cancer, found their differential expression and significant correlation with immune cell infiltration. We identified that cluster 2 gastric cancer highly expressed diverse positive regulators, thereby leading to a better prognosis and higher T cell infiltration compared with cluster 1 gastric cancer. Furthermore, various immune checkpoint genes were significantly highly-expressed in cluster 2, as well as high TMB and MSI in cluster 2. Therefore, for all gastric cancer cases, cluster 2 may benefit more from immunotherapy. Interestingly, in CTLA4- PD1- and CTLA4+ PD1-, cluster 1 showed better immunotherapy response. We preliminarily confirmed that T cell-positive regulator-related genes affect immune cell infiltration, tumor progression, and immunotherapy response. These results have instilled in us, an interest in further research.

A comprehensive network of genes is involved in gastric cancer (39). Therefore, we identified the prognostic DEGs from the positive regulator subtypes and conducted LASSO Cox regression analysis. Then, a T cell-positive regulator-related signature was identified, which showed robust capacities for predicting survival, immunotherapy response, and drug sensitivity. To further explore the mechanisms of T cell-positive regulators in gastric cancer, we performed functional enrichment analyses for putative patterns of action of T cell-positive regulators. We have identified multiple crucial signaling pathways associated with T cell-positive regulators, including the Wnt, cGMP-PKG, and cAMP signaling pathways. Goto et al., reported that in the gastric mucosa of gastric cancer infected by *Helicobacter pylori*, inducible nitric oxide synthase was highly expressed, leading to the sustained generation of nitrogen species (40). They proposed that this mechanism might account for the carcinogenesis of gastric cancer. Besides, previous literature has approved that nitric oxide produced by endothelium can induce the cyclic guanosine monophosphate (cGMP), sequentially acting cGMP-dependent protein kinase (PKG-I) (41). It has been established that the expression of PKG-I is associated with gene transcription, protein synthesis, degradation, and mRNA stability (40). Consequently, we postulate that there exists a correlation between gastric cancer, Helicobacter pylori infection, nitric oxide, and the cGMP-PKG signaling pathway. Additionally, we have observed that the Wnt signaling pathway may play a role in regulating T cell-positive regulators in gastric cancer. Previous studies have indicated that the Wnt signaling pathway serves as a pivotal mechanism for various molecules to facilitate oncogenesis and tumor progression in gastric cancer (42, 43). Interestingly, the interactions between the Wnt and PI3K-AKT signaling pathway have been identified in nearly every cancer type (44–46). Zhang et al., demonstrated that a decrease in m6A methylation provokes Wnt/PI3K-Akt signaling, thereby promoting malignant phenotypes of gastric cancer (47). Shorning et al., reported the cooperation of PI3K-AKT-mTOR and Wnt signaling pathways to facilitate the progression and drug resistance of prostate cancer (44). Additionally, in a study by Reddy et al., Lanatoside C was confirmed to induce G2/M cell cycle arrest by blocking MAPK/Wnt/PAM signaling pathways (48). Notably, in our GESA analysis, the cell cycle pathway was one of the most enriched pathways in low-risk gastric cancer, revealing that T cell-positive regulators may regulate tumorous growth by inducing cell cycle arrest through Wnt signaling pathways. In fact, with deeper exploration, a conspicuous and complicated cross-link was identified between T cell-positive regulators and the putative enriched functions, which could potentially direct future research.

Legut et al., demonstrated that the overexpression of T cell-positive regulators in T cells promotes their proliferation and function (17). According to our analysis, overexpression of T cell-positive regulators in gastric cancer cells also appeared to promote the proliferation of T cells, similar to the immune cell infiltration analysis. Although no concrete evidence has confirmed this hypothesis, our results are significant. In addition, we performed various analyses related to immunotherapy response, low-risk gastric cancer presents with higher TMB, MSI, and TIS, as well as lower TIDE. The identification of novel biomarkers has provided evidence indicating that immunotherapy may be more advantageous for individuals with low-risk gastric cancer. Moreover, low-risk gastric cancer patients with CTLA4- PD1+, CTLA4+ PD1-, and CTLA4+ PD1+ subtypes also exhibited improved response to immunotherapy. Additionally, the IC50 analysis of various drugs, including 5-fluorouracil and bleomycin, demonstrated that low-risk gastric cancer patients displayed heightened sensitivity to most drug types. Consequently, our analysis suggests that low-risk patients are better suited to receive a combination of immunotherapy and chemotherapy. Therefore, the T cell-positive regulator-related signature provides guidance for therapy selection.

According to our research, it has been determined that the T cell cluster within the tumor immune microenvironment expresses all of the signature genes. From the RT-qPCR and immunohistochemical, the expression of *DNAAF3* was higher in tumor and the expression of *PI15*, *UPK1B*, and *CGB5* was lower in tumor. Specifically, *DNAAF3* has been verified maybe an oncogene in gastric cancer, exerting an influence on both migration and proliferation capabilities. This gene, associated with cilia, encodes a cytoplasmic protein consisting of 608 amino acids, which aids in the preassembly and transportation of dynein arms from the cytoplasm to the axoneme. Additionally, *DNAAF3* has been reported to be associated with primary ciliary dyskinesia. A previous study (49) identified a high mutation rate of *DNAAF3* in patients with primary ciliary dyskinesia. However, there is currently no research available on the association between *DNAAF3* and gastric cancer or T cell. Our study presents evidence suggesting that *DNAAF3* may play a positive regulatory role in the activation of Treg cells induced by gastric cancer through the β-Catenin-induced CCL28 pathway. In our current investigation, we observed that gastric cancer cells with DNAAF3 gene knockdown exhibited reduced transwell membrane crossing ability and colony formation capacity. Moreover, the activation of T cell that co-cultured with gastric cancer cell was reduced after *DNAAF3* gene knockdown in cancer cells.

Despite the global analysis of T cell-positive regulators in gastric cancer, there are several limitations to our study. First, a prospective cohort of a large number of patients who have received immunotherapy or chemotherapy must be studied to verify the precision of our signature in the real-world scenario. Second, all of our conjectural mechanisms of T cell-positive regulators in gastric cancer are based only on functional enrichment analyses using online databases. Their detailed functions should be explored using *in vivo* and *in vitro* experiments. Furthermore, the AUC value of the predictive model in this study exhibits a range of 0.7 to 0.9, potentially attributed to the substantial heterogeneity observed in stomach cancer. It is important to note that stomach cancer encompasses diverse subtypes, whereas our signature solely pertains to whole stomach adenocarcinoma, thereby restricting the availability of more comprehensive raw data. This limitation can be addressed by incorporating more detailed data in future research endeavors. Finally, complicated TME exist in every cancer type, and the functions of T cells in diverse types of tumors cannot be neglected. However, in our study, only gastric cancer was analyzed. To overcome these limitations, we plan to design a study that contains sufficient real-world patients and explore the concrete mechanisms of T cell-positive regulators in gastric cancer. Additionally, a comprehensive pan-cancer analysis can guide future research worldwide.

## Conclusion

A T cell-positive regulator-related signature was identified in gastric cancer, demonstrating significant associations with the tumor microenvironment (TME), clinical characteristics, and response to immunotherapy. A nomogram was subsequently developed, incorporating this signature and clinical characteristics, to provide prognostic predictions for individual patients. Furthermore, an in-depth analysis was conducted to elucidate the putative molecular mechanisms underlying the role of T cell-positive regulators in gastric cancer, unveiling their potential functional patterns. This comprehensive characterization of T cell-positive regulators in gastric cancer highlights their potential as therapeutic targets, offering valuable guidance for treatment strategies. We also demonstrated that the knockdown of *DNAAF3* can reduce gastric cancer migration and proliferation and decrease the activation of T cell caused by gastric cancer in tumor microenvironment.

## Materials and methods

### Data collection

Transcriptome profiling data of 375 tumors and 32 normal tissues in fragments per kilobase million (FPKM) format, along with clinical information on gastric cancer, were obtained from The Cancer Genome Atlas (TCGA) database (https://portal.gdc.cancer.gov/). The TCGA stomach adenocarcinoma (TCGA-STAD) dataset was randomly divided into TCGA-train and TCGA-test datasets in a 1:1 ratio. To identify additional datasets for validation, the Gene Expression Omnibus (GEO) database (https://www.ncbi.nlm.nih.gov/geo/) was searched for datasets containing survival information. From this search, GSE84437 and GSE13861 were selected as the test train (50, 51). Subsequently, the GSE84437 and GSE13861 datasets were merged within the GEO database. The selection of 33 positive regulators of T cell functions utilized in this study was based on the findings of Mateusz Legut et al., who conducted a comprehensive genome-scale screening (17).

### Differential expression of genes and infiltration of immune cells, and survival analysis

Evaluation of the differential expression of T cell positive regulators and immune-related genes in our analysis were conducted with the “limma” R package (52). Moreover, along with “limma”, the immune cell infiltrations were evaluated using the “TIMER,” “CIBERSORT,” “CIVERSORT-ABS,” “QUANTISEQ,” “MCPCOUNTER,” “XCELL,” and “EPIC” algorithms (53–57). The survival analyses were performed using the Kaplan-Meier (K-M) analysis with the “survival” R package including the comparisons of different clustering subtypes and risks (58).

### Immunotherapy response and drug sensitivity

Biomarkers predicting immunotherapy responses involved in our study included the TME, TMB, MSI, TIDE scores, various immune regulatory factors, and TIS. The TME scores, including ImmuneScore, StromalScore, and ESTIMATEScore, were calculated using the “ESTIMATE” algorithm. The TMB score was calculated using the R package “maftools” (59), MSI score was obtained from a previous study (60), and the TIDE score was calculated on an online database (http://tide.dfci.harvard.edu/). In addition, we conducted a correlation analysis between the model and various immune regulatory factors, such as CCL14, CXCL6, TAP1, HLA-B, HLA-C, CCR9, among others. Moreover, we assessed the Tumor Immune Score (TIS) between low- and high-risk gastric cancer, utilizing the methodology established by Ayers et al. and Hu et al., where the TIS was determined as a weighted linear combination of scores derived from 18 specific genes (61–63).

Drug sensitivity in STAD based on the GDSC database (https://www.cancerrxgene.org/) was evaluated using the “pRRophetic” R package (64). The inhibitory concentration (IC_50_) was evaluated to determine drug sensitivity. Moreover, IMvigor210 was used to predict the correlation between the immunotherapy response and risk score (65).

### Consensus clustering

Consensus clustering of gastric cancer was performed by the “ConsensusClusterPlus” R package (66), with the gene expression of T cell positive regulators. The distance for clustering was Euclidean, repeated 1000 times.

### Establishment and verification of T cell-positive regulator-related signature

A Least absolute shrinkage and selection operator (LASSO) regression analysis was conducted with the prognostic DEGs of clusters 1 and 2 using the “glmnet” R package (67), with 1000 iterations, to identify the optimal prognostic genes. Then, a multivariate Cox regression was conducted to finally determined the model genes after the LASSO regression analysis. The signature can be calculated using the following formula:


$$\text{Risk score}=\sum_{i=1}^{n}Coef\left(i\right)*Expr\left(i\right)$$

The TCGA-train dataset was divided into low- and high-risk groups based on the median score, specifically for gastric cancer. To illustrate the relationship between survival status and risk score, scatter plots were employed across the TCGA-train, TCGA-test, and GEO datasets. Additionally, the predictive capability of the signature was assessed through survival and receiver operating characteristic (ROC) analyses conducted on the TCGA-train, TCGA-test, and GEO datasets (68). Following that, t-distributed stochastic neighbor embedding (t-SNE) and principal component analysis (PCA) were conducted to distinguish between low- and high-risk gastric cancer based on data obtained from TCGA-train, TCGA-test, and GEO datasets (69, 70).

### Construction and evaluation of the nomogram

Univariate and multivariate Cox regression analyses of risk scores and clinical characteristics across TCGA-STAD and GEO datasets were used to identify independent predictive abilities. The “rms” and “regplot” R packages were used to construct a nomogram comprising of risk, age, gender, tumor grade, and clinical, T, N, and M stages (71). Predictive probability was estimated using the calibration curve (72).

### Functional enrichment analyses

Functional enrichment analyses, including the Gene Ontology (GO) and Kyoto Encyclopedia of Genes and Genomes (KEGG) enrichment analyses, Gene Set Enrichment Analysis (GSEA), and gene set variation analysis (GSVA) were used to analyze the putative biological functions of DEG in low- and high-risk gastric cancer groups (73–75). The “c2.cp.kegg.v7.4. symbols.gmt” file downloaded from the Molecular Signatures Database (MSigDB) database (https://www.gseamsigdb.org/gsea/index.jsp). Functional analyses were conducted using the “org.Hs.eg.db,” “clusterProfiler,” “enrichplot,” and ““ GSVA R packages.

### Single-cell sequencing analysis of signature genes

Initially, we acquired single-cell RNA sequencing (scRNA-seq) data and matched bulk RNA sequencing (RNA-seq) data of gastric tumors from the Gene Expression Omnibus (GEO) database, specifically GSE212212 (76). Subsequently, t-distributed stochastic neighbor embedding (t-SNE) analysis was performed utilizing the marker genes expressed by individual cells, followed by the amalgamation of comparable categories. The expression levels of four distinct genes, characteristic of immune and stromal cell clusters, were subsequently presented.

### Cell culture and RT-qPCR

Normal stomach epithelial (GES-1) and stomach cancer (HGC-27) cells were obtained from Shanghai Institute of Cell Biology, Chinese Academy of Sciences. The cell lines were cultured in RPMI-1640 (Gibco) supplemented with 10% fetal bovine serum (FBS; Gibco), 100 U/ml penicillin, and 100 mg/ml streptomycin (Invitrogen) and incubated at 37°C with 5% CO_2_.

Cells were transfected using Lipofectamine 2000 (Invitrogen) after 6 hours of incubation. DNAAF3 inhibitors and its negative controls (NC) were synthesized from GenePharma company (Shanghai, China). Before reaching the 90% confluence point, the cells were given 24 hours of starvation for further analysis.

For Treg isolation, a Treg isolation kit (Miltenyi Biotec) was used to purify Tregs from approximately 4 × 10^7^ human CD4+T lymphocytes isolated from peripheral blood according to the manufacturer’s instructions. The Treg cells were all grown in RPMI-1640 supplemented with 10% FBS, 100 U/ml penicillin, and 100 mg/ml streptomycin, 25 μl/mL human recombinant IL2 (Stemcell), and 25 μl/mL CD3/CD28 T-cell activator (Stemcell).

Total RNA was extracted from cells using Trizol (Invitrogen). The Hifair^®^ III one-step RT-qPCR SYBR Green Kit (Yeasen, China) was used to reverse-transcribe RNA into complementary DNA (cDNA). Following the manufacturer’s instructions, RT-qPCR was conducted using Hieff^®^ qPCR SYBR Green Master Mix (Yeasen, China) (**Supplementary File S4**).

### Human sample collection and immunohistochemical

We collected 6 pairs of gastric cancer tissues and adjacent normal tissues from The First Affiliated Hospital of Ningbo University, which was approved by the ethics committees of The First Affiliated Hospital of Ningbo University (No. 2022-068A-01). The gastric cancer tissues and adjacent normal tissues were fixed with 10% formalin, embedded by paraffin, and sectioned; then we selected the optimal tissue sections for degreasing and immunohistochemistry staining. Protein expression levels were scored separately by two qualified pathologists. A positive case was defined as that in which ≥ 50% of the cancer cells had moderate staining intensity in *CGB5*. Antibody of immunohistochemical was as follow: CGB5 (Abcam, ab131170).

### Transwell migration and colony formation assays

Transwell migration assay were performed to evaluate migration after *DNAAF3* knockdown in stomach cancer cells. The cells were added to the upper chambers of 24-well transwell inserts (Corning) at a density of 2 × 10^5^ cells/well and maintained in serum-free medium for 24 h. The lower chamber was filled with 600 μL of 20% fetal bovine serum. The cells were then washed with PBS, fixed with 4% paraformaldehyde for 30 min, and stained with crystal violet.

Gastric cancer cells were cultured in 6-well plates (Corning) at a density of 1,000 cells per well for 14 days. The cells were washed with phosphate-buffered saline (PBS) (Gibco), fixed with 4% paraformaldehyde (Beyotime) for 30 min, and stained with crystal violet (Beyotime).

### Viability assessment of T cell co-cultured with gastric cancer

The T cell and gastric cancer were co-cultured in under distant transwell co-culture conditions. Viability of T cell was assessed using the Cell Counting Kit-8 (CCK-8) assay at the 0h, 24h, 48h, and 72h. Finally, WST-8 from the kit were added, and absorbance in each well was measured at 450 nm after incubation for 2 h at 37°C and results are expressed as optical densities (OD).

### Statistical analysis

Statistical analyses were conducted using R-x64-4.1.1 and Perl-5.32 (**Supplementary File S5**). Data are presented as the mean ± standard deviation. Differences were considered statistically significant at p< 0.05.

## Data availability statement

The original contributions presented in the study are included in the article/**Supplementary Material**. Further inquiries can be directed to the corresponding authors.

## Ethics statement

The studies involving humans were approved by The First Affiliated Hospital of Ningbo University Ethics Committee. The studies were conducted in accordance with the local legislation and institutional requirements. The participants provided their written informed consent to participate in this study

## Author contributions

KH, YZ, and YG designed the study and prepared draft of the manuscript. KC, YD, and YM wrote the manuscript. KH, KC, and YZ searched for publications associated with literature review and collected the data. YD and YG analyzed the data. The final manuscript was reviewed and approved for publication by all authors.

## Supplementary material

Supplementary File S1 The T cell positive regulators.

Supplementary File S2 The prognostic T cell positive regulator-related DEGs.

Supplementary File S3 The risk scores of gastric cancer patients.

Supplementary File S4 RT-qPCR sequence.

Supplementary File S5 Raw R code of the analysis.

Supplementary Figure 1
**(A)** Immune cell infiltration between cluster 1 and 2. **(B)** Expression of signature genes in tumor immune microenvironment by single-cell sequencing analysis.

Supplementary Figure 2 Verification of the T cell-positive regulator-related score in TCGA-test. **(A)** Distribution of risk score in TCGA-test. **(B)** Survival status plot and survival time of low- and high-risk gastric cancer in TCGA-test. **(C)** Survival analysis of low- and high-risk gastric cancer in TCGA-test. **(D)** Survival analysis of low- and high-risk gastric cancer in TCGA-test. **(E)** PCA analyses of low- and high-risk gastric cancer in TCGA-test. **(F)** t-SNE analysis of low- and high-risk gastric cancer in TCGA-test.

Supplementary Figure 3
**(A)** Subgroup analysis for the difference of T cell-positive regulator-related risk score in TCGA-STAD dataset. **(B)** Subgroup analysis for the difference of T cell-positive regulator-related risk score in GEO dataset. **(C)** Survival analysis of low- and high-risk gastric cancer in different subgroups. (**E**) Risk score between C1 and C2 clusters. (**D**) Immunotherapy response prediction across IMvigor210 cohort.
