# Supplementary material for: Construction and validation of a signature for T cell-positive regulators related to tumor microenvironment and heterogeneity of gastric cancer
Source: Front Immunol. 2023 Aug 30;14:1125203. doi: 10.3389/fimmu.2023.1125203 (PMC10498473; doi:10.3389/fimmu.2023.1125203)
Supplement: Supplementary File S1 — The T cell positive regulators. [file DataSheet_1.docx]

| IFNL2 |
| --- |
| LTBR |
| IL1RN |
| CXCL12 |
| CRLF2 |
| IL12B |
| NFYB |
| BATF |
| FOSB |
| ATF6B |
| AHNAK |
| SLC10A7 |
| CALML3 |
| CLIC1 |
| RAN |
| CDK2 |
| MS4A3 |
| CDK1 |
| DBI |
| CYP27A1 |
| AKR1C4 |
| DUPD1 |
| GPD1 |
| GPN3 |
| AHCY |
| ADA |
| ITM2A |
| HOMER1 |
| MRPL18 |
| MRPL51 |
| L1G3 |
| ZNF830 |
| DCLRE1B |
