# Supplementary material for: Construction and validation of a signature for T cell-positive regulators related to tumor microenvironment and heterogeneity of gastric cancer
Source: Front Immunol. 2023 Aug 30;14:1125203. doi: 10.3389/fimmu.2023.1125203 (PMC10498473; doi:10.3389/fimmu.2023.1125203)
Supplement: Supplementary File S2 — The prognostic T cell positive regulator-related DEGs. [file DataSheet_2.docx]

| id | HR | HR.95L | HR.95H | pvalue |
| --- | --- | --- | --- | --- |
| STEAP4 | 1.3716181 | 1.0923082 | 1.7223492 | 0.0065288 |
| SNORC | 0.7046362 | 0.5297661 | 0.937229 | 0.0161546 |
| CGB5 | 1.4178169 | 1.0243602 | 1.9624004 | 0.0352836 |
| PI15 | 1.4257212 | 1.1676602 | 1.7408154 | 0.0004988 |
| UPK1B | 1.1550865 | 1.0111846 | 1.3194673 | 0.0336864 |
| VSTM2L | 1.1699423 | 1.0086507 | 1.3570258 | 0.0381006 |
| DNAAF3 | 0.5521665 | 0.3197789 | 0.9534332 | 0.0330832 |
| APOD | 1.1196748 | 1.0151649 | 1.2349438 | 0.023758 |
| CST6 | 1.1874021 | 1.0145719 | 1.3896736 | 0.032337 |
| FGF7 | 1.2340535 | 1.0220206 | 1.4900756 | 0.0287852 |
| BMPR1B | 1.4981479 | 1.0746708 | 2.0884975 | 0.0170872 |
| FABP4 | 1.222187 | 1.0192199 | 1.4655728 | 0.030355 |
| VEGFD | 1.7305254 | 1.1016337 | 2.7184337 | 0.0173114 |
