# Supplementary material for: Construction and validation of a signature for T cell-positive regulators related to tumor microenvironment and heterogeneity of gastric cancer
Source: Front Immunol. 2023 Aug 30;14:1125203. doi: 10.3389/fimmu.2023.1125203 (PMC10498473; doi:10.3389/fimmu.2023.1125203)
Supplement: Supplementary File S3 — The risk scores of gastric cancer patients. [file DataSheet_3.docx]

| id | CGB5 | PI15 | UPK1B | DNAAF3 | riskScore | risk |
| --- | --- | --- | --- | --- | --- | --- |
| TCGA-BR-8682 | 0 | 0.596814953 | 0.58351581 | 0.116526306 | 0.96034865 | high |
| TCGA-R5-A7ZR | 0.033896441 | 0.261073172 | 5.08441724 | 0.093025225 | 1.701622248 | high |
| TCGA-CD-8534 | 0 | 0.589487575 | 0.086194056 | 0.358018884 | 0.786673418 | low |
| TCGA-BR-7717 | 2.023125451 | 1.396849428 | 3.955108178 | 0.321166728 | 3.75242558 | high |
| TCGA-BR-4256 | 0.092504121 | 2.321962719 | 0.615878695 | 0.203878829 | 1.71678479 | high |
| TCGA-BR-A44U | 0 | 0.665063897 | 0.094085332 | 0.16115593 | 0.893944173 | low |
| TCGA-D7-A6EZ | 0 | 0.727414715 | 0.314522058 | 1.877636682 | 0.391481812 | low |
| TCGA-RD-A8N5 | 0 | 4.521579084 | 0.555535047 | 0.085966211 | 3.710042495 | high |
| TCGA-F1-A72C | 0 | 0.588519741 | 0.387910727 | 0.816247273 | 0.650111204 | low |
| TCGA-IN-7806 | 0.136386774 | 0.881940917 | 0.135046096 | 0.404985279 | 0.895914608 | low |
| TCGA-CD-A4MG | 0 | 1.035822355 | 0.111026637 | 0.726438018 | 0.761348573 | low |
| TCGA-BR-7901 | 0.289512257 | 2.26330128 | 0.007910884 | 0.149814092 | 1.693075089 | high |
| TCGA-CD-A487 | 0.200706349 | 0.243898018 | 0.077785077 | 0.603644432 | 0.659828312 | low |
| TCGA-BR-8368 | 0.043452188 | 0.906906523 | 0.0873481 | 0.510387764 | 0.823264033 | low |
| TCGA-BR-7716 | 0 | 0.109869221 | 0.921905778 | 0.611820715 | 0.663227316 | low |
| TCGA-D7-A747 | 0.160734484 | 0.233206545 | 0.061763661 | 0.176418141 | 0.805186595 | low |
| TCGA-R5-A7ZE | 0.691780251 | 1.517789176 | 0.965364 | 0.280852412 | 1.623660294 | high |
| TCGA-VQ-A91D | 0 | 0.644181256 | 4.765688555 | 1.379166236 | 0.946268477 | low |
| TCGA-KB-A93J | 0.047330506 | 0.265264771 | 0.078200508 | 0.272753014 | 0.746919782 | low |
| TCGA-CG-5718 | 0.054721667 | 0.375831493 | 0.786228825 | 0.028133859 | 0.978357197 | high |
| TCGA-BR-4279 | 0 | 4.0004688 | 2.683990128 | 0.082458414 | 4.256402553 | high |
| TCGA-D7-A4YX | 0 | 0.10262184 | 0.054921697 | 0.424777113 | 0.640840397 | low |
| TCGA-BR-A4J5 | 1.983284776 | 0.617881838 | 0.069474177 | 0.271300645 | 1.642052533 | high |
| TCGA-BR-6452 | 0.315481138 | 0.078140459 | 1.127619414 | 1.747546807 | 0.421374299 | low |
| TCGA-VQ-A91V | 1.778188387 | 0.035388541 | 0.751368252 | 0.566092384 | 1.192096976 | high |
| TCGA-SW-A7EB | 0 | 1.055628814 | 3.652687254 | 0.692408718 | 1.314137357 | high |
| TCGA-BR-4369 | 0.128195999 | 1.197268442 | 0.520603382 | 0.453351215 | 1.02729421 | high |
| TCGA-CD-5803 | 0 | 0.364747674 | 5.491238377 | 0.136819839 | 1.80898376 | high |
| TCGA-BR-A4J8 | 0 | 0.678586765 | 0.548267141 | 0.303750827 | 0.892561924 | low |
| TCGA-BR-A44T | 0.027217053 | 0.796067589 | 0.108022094 | 0.249955458 | 0.903534875 | low |
| TCGA-HU-A4H0 | 0 | 0.020773316 | 0.006592559 | 1.474555971 | 0.361351492 | low |
| TCGA-BR-6566 | 0.078681776 | 0.118542779 | 0.087340509 | 0.394051132 | 0.675787547 | low |
| TCGA-CG-5720 | 0.026862142 | 2.347822672 | 0 | 0.348051986 | 1.436277968 | high |
| TCGA-IN-A6RL | 0.543023354 | 0.9939193 | 3.46041625 | 0.147237786 | 1.994630287 | high |
| TCGA-RD-A8MV | 0.220381718 | 1.997069798 | 0.116662022 | 0.157815572 | 1.527633686 | high |
| TCGA-VQ-A94T | 0.081414897 | 1.328021847 | 0 | 0.08290048 | 1.183594266 | high |
| TCGA-BR-8687 | 0.513191058 | 0.068292875 | 0.850953884 | 0.234306455 | 0.937245994 | low |
| TCGA-IN-A7NT | 0.368697065 | 2.677070011 | 0.993236252 | 1.748833881 | 1.021203045 | high |
| TCGA-BR-7704 | 0 | 0.819792585 | 0.037005645 | 1.057764945 | 0.59032553 | low |
| TCGA-RD-A7BS | 0.514276409 | 1.331804946 | 1.888365688 | 0.374147758 | 1.565326144 | high |
| TCGA-BR-8380 | 0.588543439 | 0.74987361 | 0.038025285 | 0.169451592 | 1.113358649 | high |
| TCGA-BR-4363 | 0.026038328 | 0.754510771 | 4.937952136 | 0.22851273 | 1.833718272 | high |
| TCGA-BR-4191 | 0.358525108 | 0.71606903 | 0.884154554 | 0.165554653 | 1.153852567 | high |
| TCGA-BR-A4PF | 0 | 0.30563147 | 0.019414855 | 0.065555355 | 0.821319659 | low |
| TCGA-VQ-A8PH | 0.051549878 | 0.089672579 | 1.142971455 | 0.121704109 | 0.890412241 | low |
| TCGA-BR-A4J6 | 0.020016121 | 0.089544509 | 5.578962255 | 2.076110278 | 0.621894254 | low |
| TCGA-VQ-AA6F | 0.019259282 | 0.203668257 | 5.597633071 | 0.520749801 | 1.438174536 | high |
| TCGA-CG-5717 | 0 | 0.905422686 | 0.929572873 | 0.312020696 | 1.015834306 | high |
| TCGA-VQ-A8DT | 0.523429473 | 0.465053352 | 1.120448161 | 0.3875754 | 1.035978327 | high |
| TCGA-D7-A4YU | 0 | 0.864956063 | 0.072959308 | 1.46678006 | 0.488737385 | low |
| TCGA-RD-A8MW | 0 | 2.420507464 | 0.058026306 | 0.520580352 | 1.346828889 | high |
| TCGA-VQ-A8PD | 2.336108254 | 1.675555816 | 1.439153953 | 0.456736562 | 2.960925069 | high |
| TCGA-BR-4201 | 0.239047255 | 2.504974222 | 4.584242777 | 1.295725375 | 1.97105219 | high |
| TCGA-RD-A8N2 | 0 | 1.879950376 | 2.176866234 | 0.032523685 | 1.964706148 | high |
| TCGA-VQ-A8P8 | 0.470775902 | 1.000111805 | 1.639815144 | 0.658764457 | 1.147294798 | high |
| TCGA-HU-A4H6 | 0.02265589 | 1.192421443 | 3.004623454 | 0.505128945 | 1.388469334 | high |
| TCGA-CG-4443 | 0 | 0.651669314 | 0.050862248 | 0.703052491 | 0.669851978 | low |
| TCGA-BR-8588 | 0 | 1.4193636 | 0.060065208 | 0.151681034 | 1.156339708 | high |
| TCGA-CD-5799 | 0 | 0.344291533 | 1.062128621 | 0.290998065 | 0.864527585 | low |
| TCGA-HU-A4GJ | 0.047556243 | 0.285212215 | 1.768388458 | 0.042468248 | 1.085483619 | high |
| TCGA-CG-4436 | 0 | 1.031496125 | 1.741021219 | 0.066828777 | 1.355130334 | high |
| TCGA-D7-8579 | 0.064736947 | 1.347316986 | 0.465092859 | 0.228281215 | 1.177513458 | high |
| TCGA-MX-A5UG | 3.448495301 | 1.619956179 | 0.903474022 | 0.200826353 | 4.492943389 | high |
| TCGA-VQ-A8E3 | 0.05155404 | 0.716751354 | 0.329460387 | 1.240066378 | 0.551670781 | low |
| TCGA-BR-A4J9 | 0.019811761 | 0.604078252 | 0.022041644 | 0.040083902 | 0.928043401 | low |
| TCGA-HU-A4GP | 0.04192379 | 0.187274689 | 0.619385589 | 0.448083721 | 0.718627664 | low |
| TCGA-VQ-A8PB | 0.018070957 | 0.753485419 | 0 | 0.610220407 | 0.726417353 | low |
| TCGA-HU-A4GY | 0.068873028 | 1.578404515 | 0.02594668 | 0.185221596 | 1.222758052 | high |
| TCGA-BR-8361 | 0 | 0.0134718 | 0.074956109 | 1.379697475 | 0.382232624 | low |
| TCGA-RD-A7BT | 0 | 0.123280795 | 0.830325266 | 0.090099673 | 0.858784426 | low |
| TCGA-VQ-A925 | 0.180439045 | 0.034494661 | 6.037387713 | 0.35694924 | 1.665156534 | high |
| TCGA-BR-8384 | 0 | 1.428823101 | 0.45857897 | 0.161587055 | 1.223983045 | high |
| TCGA-HF-7132 | 0 | 1.159985618 | 3.360843222 | 0.577716529 | 1.383460245 | high |
| TCGA-VQ-A8PC | 0.089689038 | 0.538323606 | 1.610318316 | 0.161485284 | 1.103751741 | high |
| TCGA-VQ-A91Z | 0 | 1.00143693 | 0.055964049 | 0.555028052 | 0.814900091 | low |
| TCGA-FP-7829 | 0.138287219 | 1.00354683 | 0 | 0.656713182 | 0.805249372 | low |
| TCGA-BR-A4CR | 0.019081668 | 2.1750375 | 0 | 0.192099077 | 1.462699967 | high |
| TCGA-HU-A4G2 | 0.070504327 | 0.425309979 | 0.097212962 | 0.457451092 | 0.725428306 | low |
| TCGA-CG-4460 | 2.300513273 | 1.381969943 | 1.301090665 | 0.638908018 | 2.361541645 | high |
| TCGA-CG-4465 | 0 | 0.98564615 | 0.008886356 | 0.351179169 | 0.89353432 | low |
| TCGA-BR-4370 | 0 | 1.558682013 | 0.998207637 | 0.714281131 | 1.043674512 | high |
| TCGA-HF-7134 | 0.048058112 | 0.064578419 | 0.009039928 | 0.192001077 | 0.719732434 | low |
| TCGA-IN-A6RN | 1.736607906 | 0.37809529 | 3.470695637 | 0.288123932 | 2.273499499 | high |
| TCGA-HU-A4GD | 0.446571524 | 1.848349122 | 1.01512857 | 1.291390792 | 1.002515804 | high |
| TCGA-D7-6525 | 3.398175633 | 0.114116021 | 0.225226181 | 1.125512908 | 1.487894298 | high |
| TCGA-HU-A4H2 | 0.017306113 | 0.238933445 | 0.031951769 | 0.543961628 | 0.633234438 | low |
| TCGA-IN-AB1V | 0 | 0.930074359 | 2.079797929 | 0.274910586 | 1.236835035 | high |
| TCGA-VQ-A91E | 0.019195648 | 0.222631477 | 3.181602556 | 0.796493182 | 0.880462721 | low |
| TCGA-CD-8532 | 0.025401394 | 0.170249667 | 4.064333633 | 0.538447477 | 1.12657116 | high |
| TCGA-BR-8679 | 0.612479516 | 0.318399105 | 0.030294376 | 0.120850135 | 0.992122158 | high |
| TCGA-CG-5725 | 0.321318803 | 0.093065059 | 0.675922763 | 0.385312745 | 0.797959664 | low |
| TCGA-BR-4368 | 0 | 0.528468101 | 0.273706486 | 0.307367085 | 0.81285349 | low |
| TCGA-BR-8058 | 0 | 0.219647826 | 4.063980023 | 0.368708685 | 1.238865515 | high |
| TCGA-HU-8602 | 0 | 0.73626335 | 0.015714343 | 3.04617846 | 0.206486011 | low |
| TCGA-BR-8690 | 0 | 0.315609586 | 0.174671922 | 0.198129002 | 0.787813175 | low |
| TCGA-VQ-A91X | 0 | 0.328871153 | 0.310381307 | 0.16526997 | 0.821073669 | low |
| TCGA-VQ-AA6K | 0.0692772 | 0.642254903 | 1.914152703 | 0.879249756 | 0.822137762 | low |
| TCGA-HU-A4GU | 0.020203679 | 0.251689405 | 0.007531424 | 0.319431112 | 0.711687903 | low |
| TCGA-R5-A7ZF | 0.091357717 | 1.783721072 | 0.034585063 | 1.789048114 | 0.581872731 | low |
| TCGA-D7-8576 | 1.056654968 | 0.224804404 | 3.420157769 | 0.537693555 | 1.490467284 | high |
| TCGA-BR-8286 | 0.131011474 | 0.261664036 | 0.285392039 | 1.063170877 | 0.528020451 | low |
| TCGA-CG-4476 | 0 | 2.737730236 | 3.300355562 | 0.057271474 | 3.067912856 | high |
| TCGA-BR-8683 | 0.312542969 | 1.134726287 | 0.238240244 | 0.4126725 | 1.04961983 | high |
| TCGA-BR-8291 | 0.053446726 | 2.454135086 | 0.379793166 | 0.080896115 | 1.822743624 | high |
| TCGA-BR-7723 | 0 | 0.637529659 | 0.066265521 | 0.17507526 | 0.875691181 | low |
| TCGA-CD-A4MH | 0 | 0.097742939 | 0.086481203 | 0.035244185 | 0.784734109 | low |
| TCGA-BR-8364 | 0 | 0.65456129 | 5.564893325 | 0.185369031 | 1.969313811 | high |
| TCGA-BR-8381 | 0.229687216 | 0.611021916 | 0.075020673 | 0.387017378 | 0.843789585 | low |
| TCGA-CD-8524 | 1.043693761 | 1.336669007 | 0.145972811 | 0.285945221 | 1.523421368 | high |
| TCGA-D7-8573 | 0.098166842 | 0.171457804 | 0.126265538 | 0.55656008 | 0.641068758 | low |
| TCGA-BR-8059 | 1.628833774 | 1.297774247 | 0.258039092 | 0.205320189 | 1.949096894 | high |
| TCGA-D7-6521 | 0.075493652 | 1.372345927 | 1.342120051 | 0.813197532 | 1.00516187 | high |
| TCGA-CD-5801 | 0 | 0.015031401 | 0.015818657 | 0.222124467 | 0.686048272 | low |
| TCGA-CG-5732 | 0.097962097 | 0.062175425 | 1.868331974 | 0.803742181 | 0.703238295 | low |
| TCGA-BR-8366 | 4.422649465 | 0.558088327 | 0.078519554 | 0.648226585 | 3.081187023 | high |
| TCGA-HU-A4H8 | 0.037465471 | 0.037426701 | 0.062038312 | 0.577739631 | 0.587645244 | low |
| TCGA-IN-A6RR | 0.744262124 | 1.219700728 | 3.968816987 | 0.302592325 | 2.298493919 | high |
| TCGA-VQ-A8DZ | 0.157757247 | 0.688048193 | 2.781429591 | 0.198055935 | 1.386899154 | high |
| TCGA-IN-AB1X | 0 | 1.842496579 | 2.581120301 | 2.808164459 | 0.496567781 | low |
| TCGA-3M-AB46 | 0 | 0.453749761 | 0.098868831 | 1.254058224 | 0.475440088 | low |
| TCGA-BR-7715 | 0 | 0.798421025 | 0.223187869 | 0.219120556 | 0.925643965 | low |
| TCGA-BR-4257 | 0 | 1.188062209 | 0.386230662 | 0.132473465 | 1.132210625 | high |
| TCGA-BR-4187 | 0.072608999 | 4.288082401 | 0.063088118 | 0.127006334 | 3.198925784 | high |
| TCGA-BR-8485 | 0 | 1.107750798 | 0.655764701 | 0.239665169 | 1.084946907 | high |
| TCGA-R5-A805 | 0 | 3.270478709 | 3.984191028 | 0.339027553 | 3.522760606 | high |
| TCGA-HU-A4H3 | 0 | 0.993314014 | 0.129021428 | 1.667058277 | 0.464652936 | low |
| TCGA-BR-8589 | 0 | 0.033923765 | 0.045724968 | 0.207504708 | 0.698750378 | low |
| TCGA-VQ-A8PM | 0.360323032 | 0.909222875 | 0.784955357 | 0.444985569 | 1.053261147 | high |
| TCGA-VQ-A94O | 0.618345608 | 0.378255134 | 0.36138883 | 0.388382214 | 0.928897951 | low |
| TCGA-KB-A93H | 0 | 0.17522693 | 0.035580445 | 0.126162857 | 0.7633515 | low |
| TCGA-VQ-A923 | 0.049020804 | 0.550988504 | 0.422155588 | 0.900785445 | 0.628270951 | low |
| TCGA-CD-8526 | 0.091153805 | 1.621638842 | 1.668376953 | 1.693435661 | 0.735442118 | low |
| TCGA-SW-A7EA | 0.356692043 | 0.146827762 | 0.303603669 | 1.322045237 | 0.481946167 | low |
| TCGA-RD-A7C1 | 0.161387891 | 1.820360348 | 0.671782492 | 0.55313103 | 1.248889499 | high |
| TCGA-D7-A6F0 | 0.038957719 | 0.195039118 | 0.16594756 | 1.349156898 | 0.424328945 | low |
| TCGA-VQ-AA6G | 0.057817005 | 0.743619316 | 1.260726653 | 0.160957739 | 1.112477265 | high |
| TCGA-BR-4361 | 0.082048978 | 0.70526144 | 0.240000291 | 0.40374436 | 0.841236179 | low |
| TCGA-BR-8367 | 0.025419099 | 1.071017107 | 0.268446611 | 0.341540406 | 0.969005083 | high |
| TCGA-FP-A9TM | 0.056864896 | 0.272489914 | 0.779217437 | 0.581700537 | 0.711000299 | low |
| TCGA-VQ-A91K | 0.052867525 | 0.152021906 | 0.019847963 | 0.507362744 | 0.632959013 | low |
| TCGA-HJ-7597 | 0.092633187 | 0.496829943 | 1.628546385 | 2.073508358 | 0.410081695 | low |
| TCGA-R5-A7ZI | 0 | 0.085171163 | 0 | 0.314187215 | 0.668739941 | low |
| TCGA-BR-8676 | 0 | 0.031752565 | 0 | 0.322825288 | 0.653754325 | low |
| TCGA-BR-A4J7 | 0.085922633 | 0.145059165 | 0.241478031 | 0.149789254 | 0.792571792 | low |
| TCGA-BR-A4QL | 0 | 0.9284979 | 0.044290367 | 0.473699334 | 0.827255442 | low |
| TCGA-BR-8372 | 0.088418465 | 0.314135114 | 0.113835256 | 0.933209728 | 0.552069335 | low |
| TCGA-BR-8686 | 0 | 0.732556277 | 0.334748916 | 0.504040249 | 0.795087569 | low |
| TCGA-VQ-A91S | 0 | 0.343120904 | 0.544057707 | 0.71552011 | 0.644173784 | low |
| TCGA-BR-8373 | 0 | 1.04373645 | 0.124183435 | 0.222704669 | 0.990118814 | high |
| TCGA-VQ-A91U | 0 | 0.759996827 | 1.075043778 | 0.144100812 | 1.076292181 | high |
| TCGA-HU-8244 | 0.032146585 | 0.026497922 | 0.115867607 | 0.311541246 | 0.675109975 | low |
| TCGA-BR-7957 | 0.020136828 | 2.371818942 | 0.267172727 | 0.452836514 | 1.424173138 | high |
| TCGA-HU-A4HD | 0.26156367 | 1.943032375 | 0.054957319 | 0.071251115 | 1.575767423 | high |
| TCGA-VQ-A91Y | 0.484516199 | 0.958389254 | 0.289470117 | 0.096232945 | 1.242631904 | high |
| TCGA-HU-A4G9 | 0 | 0.047253256 | 0.144239064 | 1.054885976 | 0.461364539 | low |
| TCGA-HF-A5NB | 0 | 0.027972022 | 0.011844209 | 1.940003204 | 0.285603622 | low |
| TCGA-VQ-A8PO | 0 | 0.668117218 | 0.084330635 | 0.770947585 | 0.653808791 | low |
| TCGA-VQ-A8DV | 0.442384298 | 0.504475887 | 0.986668395 | 0.165895221 | 1.121561065 | high |
| TCGA-CD-A48A | 0.114268239 | 1.163457512 | 0.350862457 | 0.526931265 | 0.949214276 | high |
| TCGA-CG-4301 | 0.023107675 | 1.439132671 | 0.796651574 | 0.558672218 | 1.061778339 | high |
| TCGA-CD-A486 | 0.129033988 | 0.961399665 | 0.618938217 | 0.783828082 | 0.812084097 | low |
| TCGA-CG-5734 | 0.107339733 | 1.044094707 | 3.402544012 | 0.223072677 | 1.665126421 | high |
| TCGA-CG-4469 | 0 | 0.952157599 | 0.091011996 | 0.194074693 | 0.969074863 | high |
| TCGA-CG-5722 | 0.044384494 | 0.308789088 | 1.9758752 | 0.232738844 | 1.022298862 | high |
| TCGA-D7-8575 | 0.301241241 | 0.009056994 | 4.496429752 | 0.306337027 | 1.407586675 | high |
| TCGA-CG-4437 | 0.033477061 | 0.421536571 | 0.242411902 | 0.54635903 | 0.698218998 | low |
| TCGA-HU-A4H4 | 0.0351115 | 1.122305621 | 0.173776276 | 0.493495679 | 0.902640433 | low |
| TCGA-BR-6457 | 0 | 0.906157828 | 0.049856842 | 0.269159691 | 0.91242378 | low |
| TCGA-BR-6803 | 0 | 0.165190845 | 3.293301467 | 0.19207925 | 1.188378325 | high |
| TCGA-CD-5804 | 0 | 0.501708474 | 0.832118913 | 0.345272597 | 0.857688816 | low |
| TCGA-BR-8592 | 0.035974435 | 0.697474131 | 0.141537294 | 0.158019237 | 0.923080018 | low |
| TCGA-D7-6520 | 0 | 0.148786672 | 2.323974692 | 0.220087339 | 1.009916621 | high |
| TCGA-BR-6707 | 0 | 0.029917428 | 0.110237318 | 0.485024878 | 0.611085514 | low |
| TCGA-BR-8371 | 0.096477094 | 3.669764937 | 0.054503494 | 0.098228286 | 2.64700335 | high |
| TCGA-HU-A4G8 | 0 | 0.874756212 | 2.236949616 | 0.403637703 | 1.162835232 | high |
| TCGA-BR-4371 | 0.029561837 | 0.020908108 | 0.05438549 | 0.22493711 | 0.697423256 | low |
| TCGA-CG-4306 | 0.68639833 | 2.639483397 | 3.634860409 | 0.273071943 | 3.535874499 | high |
| TCGA-CD-8528 | 0.340416653 | 0.261461612 | 0.024929086 | 0.281312966 | 0.815287639 | low |
| TCGA-VQ-AA69 | 0.120331235 | 0.353569008 | 0 | 0.112677263 | 0.847075379 | low |
| TCGA-VQ-A92D | 0.604148107 | 0.99102781 | 0.014582478 | 1.214827326 | 0.70895327 | low |
| TCGA-BR-8295 | 0.078063256 | 0.324995349 | 0.65160062 | 0.959077347 | 0.589787432 | low |
| TCGA-VQ-A91Q | 0.039702037 | 3.128222452 | 0.277727954 | 1.076279285 | 1.350822439 | high |
| TCGA-BR-8296 | 0.064108347 | 0.622322886 | 2.93917391 | 0.725847147 | 1.025454793 | high |
| TCGA-CG-4477 | 0.031484127 | 0.91790831 | 3.272525058 | 1.004219477 | 1.021496973 | high |
| TCGA-BR-7722 | 0 | 0.180874246 | 5.935102094 | 0.281624786 | 1.684000215 | high |
| TCGA-BR-6453 | 0.070072147 | 0.865359122 | 0.02640527 | 0.080032196 | 1.012115103 | high |
| TCGA-FP-8099 | 0.068512191 | 0.349767455 | 0.17934314 | 0.338287941 | 0.760088816 | low |
| TCGA-MX-A666 | 0.118764594 | 1.283303681 | 0.636088993 | 0.653127357 | 0.968216216 | high |
| TCGA-CG-5716 | 0.037607764 | 0.94249206 | 3.164943119 | 0.057082171 | 1.650711768 | high |
| TCGA-CD-8527 | 0.032683363 | 0.300512687 | 1.612559156 | 1.510934078 | 0.499963746 | low |
| TCGA-CD-A48C | 1.842684029 | 0.36324185 | 1.355831005 | 0.223203812 | 1.776440697 | high |
| TCGA-BR-8369 | 2.360547166 | 0.899661501 | 1.398558331 | 0.378876135 | 2.370133052 | high |
| TCGA-BR-8081 | 0.04521784 | 0.566401936 | 0.168738298 | 0.731964953 | 0.662548352 | low |
| TCGA-BR-4294 | 0 | 0.266408528 | 0.047339943 | 0.168149073 | 0.77206527 | low |
| TCGA-BR-8284 | 0.044742199 | 0.396717603 | 0.323478793 | 0.457383637 | 0.736179205 | low |
| TCGA-BR-4267 | 0.016606326 | 0.12765969 | 5.686121455 | 0.130082263 | 1.732742819 | high |
| TCGA-CG-5724 | 7.819273483 | 0.750106084 | 3.420999858 | 0.910775635 | 15.18769639 | high |
| TCGA-CD-5800 | 1.075847491 | 0.150281899 | 0.23558407 | 1.4816823 | 0.564142782 | low |
| TCGA-HU-A4HB | 0.12101422 | 0.397327752 | 2.893344735 | 0.316308659 | 1.18653122 | high |
| TCGA-BR-7197 | 0 | 0.229490468 | 0.020260995 | 0.133637105 | 0.772907194 | low |
| TCGA-BR-8060 | 0.675303062 | 2.272838694 | 0.021152262 | 0.071321104 | 2.023980844 | high |
| TCGA-FP-8211 | 0.045699404 | 0.050929361 | 0.509980046 | 0.326658143 | 0.719240298 | low |
| TCGA-BR-8590 | 4.957788798 | 0.689123432 | 0.843187771 | 0.253764225 | 5.309023022 | high |
| TCGA-HF-7133 | 0.523596681 | 0.393085337 | 1.893856875 | 0.827816586 | 0.90409998 | low |
| TCGA-VQ-A8PE | 0.059104446 | 1.065368266 | 0.649926542 | 0.074854874 | 1.186531768 | high |
| TCGA-HU-A4GT | 0.023432142 | 1.148100451 | 0.060103513 | 1.631225841 | 0.497914915 | low |
| TCGA-VQ-A8PX | 0 | 0.022368154 | 0.103016117 | 0.296982878 | 0.670444858 | low |
| TCGA-VQ-A94P | 0 | 1.623681949 | 0.007597486 | 0.100935947 | 1.262745712 | high |
| TCGA-BR-6564 | 0 | 0.158296435 | 1.018232499 | 0.380273409 | 0.770065334 | low |
| TCGA-D7-6522 | 0 | 0.402529223 | 0.191079355 | 0.103421124 | 0.85395442 | low |
| TCGA-IN-A6RI | 0.07562659 | 0.697733971 | 0.47110139 | 0.328686444 | 0.90016871 | low |
| TCGA-VQ-A94R | 0.022139677 | 0.821267748 | 0.270971281 | 0.511345676 | 0.815042997 | low |
| TCGA-BR-8680 | 0.055661851 | 0.109145426 | 0.020909686 | 0.972167234 | 0.492128241 | low |
| TCGA-VQ-A8P5 | 0.136956075 | 0.872581678 | 0.575028024 | 0.931101778 | 0.727845247 | low |
| TCGA-VQ-A8PU | 0.034920167 | 0.020622014 | 2.560982929 | 0.405428237 | 0.921368745 | low |
| TCGA-D7-8574 | 0 | 0.497532354 | 0.067865524 | 0.114543976 | 0.861320075 | low |
| TCGA-D7-6822 | 0.11039338 | 0.265279655 | 0.716686042 | 1.30712888 | 0.493629989 | low |
| TCGA-HU-8608 | 0 | 0.453668965 | 0.080347822 | 1.950187687 | 0.331893811 | low |
| TCGA-BR-7707 | 0 | 0.285651258 | 0.063366163 | 1.497815184 | 0.394148959 | low |
| TCGA-VQ-A8E7 | 0.269502754 | 2.396129067 | 0.389964214 | 0.65916731 | 1.433747039 | high |
| TCGA-CG-5719 | 0.436777812 | 1.376104755 | 4.258178185 | 0.317475537 | 2.258194278 | high |
| TCGA-CD-A489 | 2.402410305 | 0.754468696 | 0.148580788 | 0.130001752 | 2.16241166 | high |
| TCGA-FP-7916 | 0 | 0.319851197 | 0.018419791 | 0.736177437 | 0.58523844 | low |
| TCGA-KB-A93G | 0 | 6.293726001 | 0.274154413 | 0.147588697 | 6.315836154 | high |
| TCGA-BR-4253 | 0.026452411 | 0.501181284 | 0.228595475 | 0.689468785 | 0.663774221 | low |
| TCGA-BR-8484 | 0 | 0.169617194 | 0.36406855 | 0.231203083 | 0.757760495 | low |
| TCGA-VQ-A8P2 | 0.030317459 | 0.271609617 | 0.011326459 | 1.897659424 | 0.320494292 | low |
| TCGA-VQ-A94U | 0.049318694 | 1.899226298 | 0.215783697 | 1.172042605 | 0.840504347 | low |
| TCGA-CG-4438 | 0.271567309 | 0.628846276 | 0.095355451 | 0.240864922 | 0.931052032 | low |
| TCGA-IN-A6RJ | 0 | 0.17936378 | 0.548792988 | 0.281163857 | 0.761511151 | low |
| TCGA-IN-7808 | 0 | 1.136713104 | 0.082753648 | 0.296186258 | 0.978300688 | high |
| TCGA-BR-6852 | 0 | 0.837004923 | 0.04959631 | 1.349431522 | 0.512328612 | low |
| TCGA-BR-7851 | 0 | 0.680339211 | 0.026156378 | 1.491676628 | 0.449953884 | low |
| TCGA-VQ-A927 | 0.099169521 | 1.84746702 | 6.002646044 | 0.341777438 | 3.014729837 | high |
| TCGA-CG-4462 | 1.859243646 | 5.247234854 | 3.745353518 | 0.244678736 | 13.3128615 | high |
| TCGA-CG-4440 | 0.550916716 | 1.314111789 | 3.6755032 | 0.786457438 | 1.659751387 | high |
| TCGA-BR-6709 | 0 | 0.561692037 | 0.014105064 | 0.289829627 | 0.79842666 | low |
| TCGA-HU-8238 | 0.358752615 | 2.51942264 | 1.282127805 | 0.480755452 | 1.927055922 | high |
| TCGA-CG-4444 | 0 | 0.929111825 | 0.032754881 | 0.293813643 | 0.905774765 | low |
| TCGA-VQ-A8E2 | 0.022786317 | 1.644897786 | 1.966036515 | 1.217769842 | 0.965244276 | high |
| TCGA-BR-4366 | 0.781434333 | 0.585779412 | 0.304544101 | 0.398588428 | 1.040559222 | high |
| TCGA-B7-A5TI | 0 | 0.946992355 | 2.195967383 | 0.404657167 | 1.184078685 | high |
| TCGA-CG-4305 | 1.803502734 | 0.476229659 | 1.571944697 | 0.656011379 | 1.506436348 | high |
| TCGA-CG-4475 | 0.023054291 | 3.879278383 | 0.034094222 | 0.749362464 | 1.979819598 | high |
| TCGA-VQ-A8PK | 0.218895091 | 1.451603076 | 5.468862511 | 0.170166969 | 2.770335327 | high |
| TCGA-D7-A6EV | 0 | 1.278504024 | 1.126069568 | 0.251582647 | 1.225063897 | high |
| TCGA-D7-5577 | 2.576256067 | 0.171278832 | 0.052459091 | 0.937233794 | 1.226713945 | high |
| TCGA-ZA-A8F6 | 0.050365616 | 0.453664436 | 0.118633646 | 0.177860019 | 0.842102642 | low |
| TCGA-RD-A8N4 | 0.020429219 | 1.498350448 | 0.04510359 | 0.271470737 | 1.122653828 | high |
| TCGA-BR-6565 | 0.078208188 | 0.258704036 | 0.019749033 | 0.05357665 | 0.835523905 | low |
| TCGA-BR-4367 | 1.743405098 | 2.702078576 | 0.116490468 | 0.317973851 | 3.027607395 | high |
| TCGA-ZQ-A9CR | 0.141327757 | 4.576699894 | 0.054080891 | 0.073718933 | 3.710016585 | high |
| TCGA-FP-A4BF | 0.921701918 | 0.587056639 | 1.36377719 | 0.962812256 | 0.956487552 | high |
| TCGA-D7-6527 | 0.101020746 | 1.31592034 | 0 | 0.233272207 | 1.098717265 | high |
| TCGA-VQ-A8PQ | 0.021570835 | 0.414496243 | 0.202829423 | 0.316784327 | 0.775739553 | low |
| TCGA-MX-A5UJ | 0.043865797 | 1.83323541 | 0.080370774 | 0.707501291 | 1.020103381 | high |
| TCGA-HU-A4GC | 0 | 0.660079777 | 6.125190888 | 0.039509501 | 2.309030361 | high |
| TCGA-BR-6563 | 0.049519446 | 0.731822058 | 0.781814253 | 0.093160777 | 1.065982692 | high |
| TCGA-VQ-AA6A | 0 | 0.647405144 | 0.146913763 | 0.259982284 | 0.851296571 | low |
| TCGA-CD-8529 | 0.070076738 | 0.584148014 | 2.651484808 | 0.635570717 | 1.018206474 | high |
| TCGA-HU-8249 | 0 | 1.944835962 | 0.309846844 | 0.272921144 | 1.348959989 | high |
| TCGA-BR-A4IV | 0 | 3.214868969 | 1.033547751 | 0.166382783 | 2.445180595 | high |
| TCGA-HU-A4H5 | 0.018741083 | 0.823629212 | 0.290686967 | 0.429037431 | 0.852309835 | low |
| TCGA-CG-5726 | 0.030091919 | 0.035301118 | 0.087572047 | 0.67434666 | 0.559541081 | low |
| TCGA-FP-8210 | 0.332140539 | 1.024627478 | 2.339942047 | 0.277311729 | 1.48662849 | high |
| TCGA-VQ-AA68 | 0 | 0.169747635 | 3.899048954 | 0.546329788 | 1.08530459 | high |
| TCGA-BR-4280 | 0 | 0.243305925 | 0.066094813 | 0.389174698 | 0.685877345 | low |
| TCGA-VQ-AA64 | 0.018062024 | 1.681485698 | 0.110346444 | 1.373802425 | 0.685381358 | low |
| TCGA-VQ-AA6J | 0 | 0.619760812 | 0.172053874 | 0.341141851 | 0.811952798 | low |
| TCGA-VQ-A928 | 0.078950017 | 3.389056134 | 0.022413467 | 0.351380924 | 2.089890076 | high |
| TCGA-BR-8080 | 0.489495873 | 2.378320176 | 0.393389071 | 0.492481333 | 1.6753398 | high |
| TCGA-IN-A7NR | 4.659159065 | 0.379107001 | 3.45887345 | 0.352933626 | 6.019405523 | high |
| TCGA-HU-A4GF | 0.150572284 | 0.691569093 | 0.10544338 | 0.094973365 | 0.984586554 | high |
| TCGA-RD-A8N6 | 0.601738347 | 1.072057767 | 0.080827039 | 0.560260556 | 1.028395563 | high |
| TCGA-VQ-A922 | 0.355992432 | 1.526984232 | 0.603449312 | 1.745127593 | 0.649489746 | low |
| TCGA-CD-5813 | 0 | 1.980954981 | 0 | 0.948056557 | 0.923222254 | low |
| TCGA-BR-8486 | 0.102481356 | 0.530778428 | 0.128157414 | 0.467283446 | 0.760003138 | low |
| TCGA-IP-7968 | 0.203043375 | 1.258091329 | 0.483070116 | 1.158139225 | 0.745933635 | low |
| TCGA-CG-5723 | 0 | 1.705977902 | 0.306016733 | 1.775930012 | 0.575299732 | low |
| TCGA-BR-A4CS | 0 | 2.323077465 | 0.05846977 | 0.139300003 | 1.583928044 | high |
| TCGA-FP-8209 | 0 | 1.635170696 | 0.055800529 | 0.094448195 | 1.280991952 | high |
| TCGA-BR-6801 | 0.055168536 | 0.234346313 | 0.061294371 | 0.136560178 | 0.792661184 | low |
| TCGA-B7-A5TN | 0.020148921 | 0.40846025 | 3.665280198 | 0.555079113 | 1.140420716 | high |
| TCGA-BR-4357 | 0.034154442 | 1.58852732 | 0 | 1.662689052 | 0.566525851 | low |
| TCGA-VQ-A8PJ | 0.088899593 | 0.828103309 | 6.864088311 | 0.271672598 | 2.496133463 | high |
| TCGA-VQ-A8PF | 0 | 1.782428276 | 0.123799191 | 0.478739506 | 1.117427953 | high |
| TCGA-F1-6177 | 0.024345582 | 0.08950032 | 0.018110365 | 0.782004973 | 0.5328542 | low |
| TCGA-BR-8382 | 0.060481663 | 0.329541444 | 0.045135037 | 0.360100606 | 0.729833212 | low |
| TCGA-BR-8077 | 0 | 0.675057312 | 0.17727183 | 0.03014007 | 0.97110558 | high |
| TCGA-BR-7196 | 0 | 1.530448851 | 0.11183746 | 0.408468837 | 1.061032727 | high |
| TCGA-EQ-8122 | 0.180951879 | 1.683134087 | 0.476959376 | 0.358949939 | 1.287817829 | high |
| TCGA-FP-7998 | 0 | 0.617962026 | 0.156651477 | 0.757796887 | 0.653982944 | low |
| TCGA-D7-6524 | 1.119731329 | 4.525162459 | 3.46988858 | 0.422522587 | 7.066625931 | high |
| TCGA-HU-A4GQ | 0.025519029 | 0.513845135 | 0.575430453 | 0.490082201 | 0.776802913 | low |
| TCGA-KB-A6F7 | 0.234367161 | 0.370926001 | 3.828357395 | 0.686796499 | 1.160695715 | high |
| TCGA-VQ-A8E0 | 0.058174304 | 0.40490127 | 0.84735733 | 0.547074472 | 0.765178299 | low |
| TCGA-BR-8483 | 0.399918968 | 0.081364172 | 0.319486144 | 0.365637336 | 0.782709653 | low |
| TCGA-FP-8631 | 0.045684113 | 0.758669284 | 0.466856471 | 0.44418213 | 0.856843621 | low |
| TCGA-D7-6519 | 3.465692426 | 0.186462271 | 1.761277187 | 1.120373176 | 1.962524035 | high |
| TCGA-D7-6815 | 0 | 0.105377427 | 0.083825724 | 0.612730405 | 0.585038901 | low |
| TCGA-CD-8530 | 0 | 2.407387016 | 0.744001658 | 0.189974686 | 1.757161335 | high |
| TCGA-BR-6456 | 0.022885222 | 0.468157249 | 0 | 1.056016049 | 0.525296107 | low |
| TCGA-MX-A663 | 0.939132075 | 0.998385442 | 1.058886533 | 0.444768084 | 1.380544555 | high |
| TCGA-FP-A8CX | 0 | 0.49399106 | 1.458704317 | 0.103169309 | 1.062032768 | high |
| TCGA-CD-8535 | 0.670316086 | 0.954815847 | 0.051835959 | 0.478522287 | 1.050473031 | high |
| TCGA-D7-A6F2 | 0.024664636 | 0.199039069 | 0.435072743 | 0.068064263 | 0.848072262 | low |
| TCGA-BR-7959 | 0.01354099 | 2.469448674 | 0.193449553 | 0.410389845 | 1.485182077 | high |
| TCGA-RD-A8NB | 0.316317117 | 0.93947221 | 0.037024235 | 0.306112187 | 1.008060718 | high |
| TCGA-B7-A5TJ | 0.807556143 | 0.283747572 | 0.054991135 | 0.062726646 | 1.084348199 | high |
| TCGA-CG-4442 | 1.021744789 | 0.852511083 | 1.606313825 | 0.470522601 | 1.445723649 | high |
| TCGA-BR-8678 | 0.047136039 | 0.792310315 | 0.607733017 | 0.378832643 | 0.915503288 | low |
| TCGA-HU-8604 | 0 | 1.082611311 | 0.04017937 | 0.16703094 | 1.019685556 | high |
| TCGA-F1-A448 | 0.085119605 | 4.23685076 | 0.548340047 | 0.354593997 | 3.017730107 | high |
| TCGA-BR-6458 | 0.150374064 | 0.846896111 | 0.113093013 | 0.272442854 | 0.948974737 | high |
| TCGA-BR-8677 | 0 | 1.423343664 | 0.617938525 | 1.003642531 | 0.812429798 | low |
| TCGA-HU-A4G3 | 0.063062957 | 0.085782321 | 3.199509341 | 0.707958506 | 0.89503144 | low |
| TCGA-HU-A4GH | 0.365804836 | 0.690049972 | 0.347370511 | 0.069019065 | 1.113082873 | high |
| TCGA-BR-8365 | 0.067646096 | 0.613839489 | 0.328114821 | 0.27730542 | 0.876951468 | low |
| TCGA-D7-A74A | 0.144193765 | 0.043880813 | 2.398282777 | 0.940474543 | 0.715798641 | low |
| TCGA-F1-6874 | 0.085657508 | 3.781718507 | 0.115358752 | 1.40679569 | 1.41399773 | high |
| TCGA-BR-6802 | 1.319202868 | 0.486717256 | 0.289223861 | 0.941378369 | 0.91494075 | low |
| TCGA-BR-7958 | 0 | 0.843529424 | 0.041014362 | 0.042201754 | 1.001951246 | high |
| TCGA-FP-7735 | 0 | 0.617558596 | 0.23696804 | 0.699889081 | 0.681600033 | low |
| TCGA-CD-8525 | 0.554597692 | 0.343197785 | 0.919621995 | 0.181819934 | 1.083653184 | high |
| TCGA-CD-8531 | 0.026476604 | 0.381356032 | 0.019699324 | 0.013547583 | 0.873570453 | low |
| TCGA-BR-6455 | 0.481834272 | 0.157779362 | 0.074476308 | 0.331747173 | 0.811097512 | low |
| TCGA-CG-5721 | 0.212974793 | 0.387881288 | 0.010607841 | 1.916599698 | 0.351695999 | low |
| TCGA-D7-6818 | 0.530807188 | 0.873044291 | 0.042659409 | 0.342251839 | 1.042540787 | high |
| TCGA-VQ-A8DU | 0.198611576 | 1.142541527 | 1.8705474 | 0.04668979 | 1.552282617 | high |
| TCGA-B7-A5TK | 0.305819621 | 0.523119495 | 0.007880216 | 0.771148623 | 0.68365558 | low |
| TCGA-VQ-A924 | 0 | 0.21726472 | 0.01364778 | 0.940879369 | 0.508472249 | low |
| TCGA-IN-8663 | 6.881139663 | 0.388454407 | 0.027520653 | 0.632907234 | 6.79310315 | high |
| TCGA-BR-A4J4 | 0 | 0.591942084 | 0.45605691 | 0.490797453 | 0.776733382 | low |
| TCGA-BR-6705 | 1.248697555 | 1.425604319 | 0.091133489 | 0.376647345 | 1.595996724 | high |
| TCGA-F1-6875 | 0.207580296 | 2.008288593 | 0 | 0.144181624 | 1.511248653 | high |
| TCGA-D7-5578 | 0.128381518 | 1.938408882 | 0.130913505 | 0.278974364 | 1.36611985 | high |
| TCGA-D7-A6EX | 0.173266642 | 1.880479831 | 0.022597703 | 0.282943489 | 1.336019463 | high |
| TCGA-HU-8610 | 0.400729948 | 2.840666254 | 0.066994082 | 0.031036355 | 2.296980873 | high |
| TCGA-D7-A4Z0 | 0.924977781 | 1.101638647 | 1.100935947 | 0.109121791 | 1.700653598 | high |
| TCGA-BR-8591 | 0.042965518 | 0.824505721 | 0.047758664 | 1.028141938 | 0.610249393 | low |
| TCGA-BR-8297 | 0.439925314 | 0.329114524 | 1.343117264 | 0.339634886 | 1.01763962 | high |
| TCGA-D7-8570 | 0 | 0.639641803 | 0.064676846 | 0.153643356 | 0.885791423 | low |
| TCGA-VQ-A91A | 0.019264948 | 5.272865262 | 0.097407214 | 0.104772449 | 4.468603164 | high |
| TCGA-BR-6710 | 0 | 0.214807685 | 2.129599551 | 0.106182839 | 1.064110529 | high |
| TCGA-VQ-AA6D | 0 | 1.194115427 | 0.147202222 | 0.185999584 | 1.065674268 | high |
| TCGA-BR-8487 | 0 | 0.134143183 | 0.217114303 | 0.946211471 | 0.5078917 | low |
| TCGA-RD-A8N9 | 0.769825554 | 2.4059518 | 0.040246253 | 0.255966792 | 1.996368278 | high |
| TCGA-BR-6454 | 0.039219758 | 0.068437911 | 0.408483837 | 0.144415417 | 0.780834093 | low |
| TCGA-D7-8578 | 1.513485187 | 0.568661498 | 4.15698791 | 0.742009057 | 1.969806196 | high |
| TCGA-IN-A7NU | 6.412169097 | 1.298285865 | 1.935793132 | 0.488999459 | 11.24072077 | high |
| TCGA-VQ-A91N | 0.408027791 | 1.046852824 | 0.570712879 | 0.466310645 | 1.075575823 | high |
| TCGA-R5-A7O7 | 0 | 1.735205876 | 0.345587359 | 0.489821686 | 1.129636676 | high |
| TCGA-D7-6526 | 0.023152795 | 1.407192292 | 0.051059026 | 0.080910478 | 1.202046376 | high |
| TCGA-IN-A6RS | 0 | 2.910234539 | 0.009540717 | 0.213335336 | 1.850084571 | high |
| TCGA-CD-5798 | 0.028313051 | 0.206301887 | 0.234542768 | 0.228509898 | 0.761250209 | low |
| TCGA-D7-6528 | 0.39154822 | 0.61862301 | 0.03663108 | 0.68205797 | 0.764726165 | low |
| TCGA-VQ-A8PP | 1.330143601 | 0.562106313 | 0.16727956 | 0.709079643 | 1.042558057 | high |
| TCGA-B7-5818 | 0 | 0.103172721 | 0.181138053 | 0.939641608 | 0.501579322 | low |
| TCGA-IN-8462 | 0.171668006 | 0.821518795 | 4.460591866 | 0.735624679 | 1.41811736 | high |
| TCGA-D7-8572 | 0.278141442 | 1.089960444 | 0.219596657 | 0.454245815 | 0.997233237 | high |
| TCGA-HU-A4GX | 0 | 0.231308533 | 1.24832048 | 1.29586288 | 0.510902501 | low |
| TCGA-D7-A748 | 2.546703363 | 0.791809738 | 4.245963352 | 0.70397943 | 3.136688496 | high |
| TCGA-RD-A7BW | 0.094054542 | 1.102977202 | 0.536088818 | 0.397711415 | 1.013840882 | high |
| TCGA-CG-4466 | 0 | 2.255459642 | 0.16063575 | 0.179875468 | 1.538919947 | high |
| TCGA-D7-A6EY | 0.890574788 | 0.39628994 | 0.756427606 | 0.264446954 | 1.159517024 | high |
| TCGA-RD-A8N1 | 0 | 0.762798246 | 0.34560791 | 0.231155026 | 0.925369102 | low |
| TCGA-BR-8289 | 0.283276059 | 0.339676499 | 0.37146162 | 1.151962048 | 0.553069742 | low |
| TCGA-CD-8533 | 0.12193684 | 0.771371693 | 0.28218416 | 0.323753046 | 0.914522664 | low |
| TCGA-VQ-A8P3 | 0 | 0.314234093 | 0.01899019 | 1.442834709 | 0.406718493 | low |
| TCGA-RD-A8N0 | 0.027275861 | 0.312956471 | 3.058574703 | 0.232660388 | 1.193731314 | high |
| TCGA-CG-4441 | 0.110738776 | 0.62650231 | 6.310006495 | 0.52497315 | 1.90038507 | high |
