# Supplementary material for: Construction and validation of a signature for T cell-positive regulators related to tumor microenvironment and heterogeneity of gastric cancer
Source: Front Immunol. 2023 Aug 30;14:1125203. doi: 10.3389/fimmu.2023.1125203 (PMC10498473; doi:10.3389/fimmu.2023.1125203)
Supplement: Supplementary File S4 — RT-qPCR sequence. [file DataSheet_4.docx]

| ID | Primer sequence(5'to3')（F） | Primer sequence(5'to3')（R） |
| --- | --- | --- |
| β-actin | TGACGTGGACATCCGCAAAG | CTGGAAGGTGGACAGCGAGG |
| PI15 | CCAGGATTGCAACCCCAGAT | TGCGCATCCTATCCGATTGG |
| DNAAF3 | GGGCTCAAGTCATTCACCCC | GGTTGGGCACATGATAGGC |
| UPK1B | CCAAAGACAACTCAACTGTTCGT | AATGCCGCAACAACCAATAATC |
| β-catenin | AAAGCGGCTGTTAGTCACTGG | CGAGTCATTGCATACTGTCCAT |
| FOXP3 | GTGGCCCGGATGTGAGAAG | GGAGCCCTTGTCGGATGATG |
| CCL28 | TGCACGGAGGTTTCACATCAT | TTGGCAGCTTGCACTTTCATC |
