# Supplementary figures and images for: Construction and validation of a signature for T cell-positive regulators related to tumor microenvironment and heterogeneity of gastric cancer
Source: Front Immunol. 2023 Aug 30;14:1125203. doi: 10.3389/fimmu.2023.1125203 (PMC10498473; doi:10.3389/fimmu.2023.1125203)

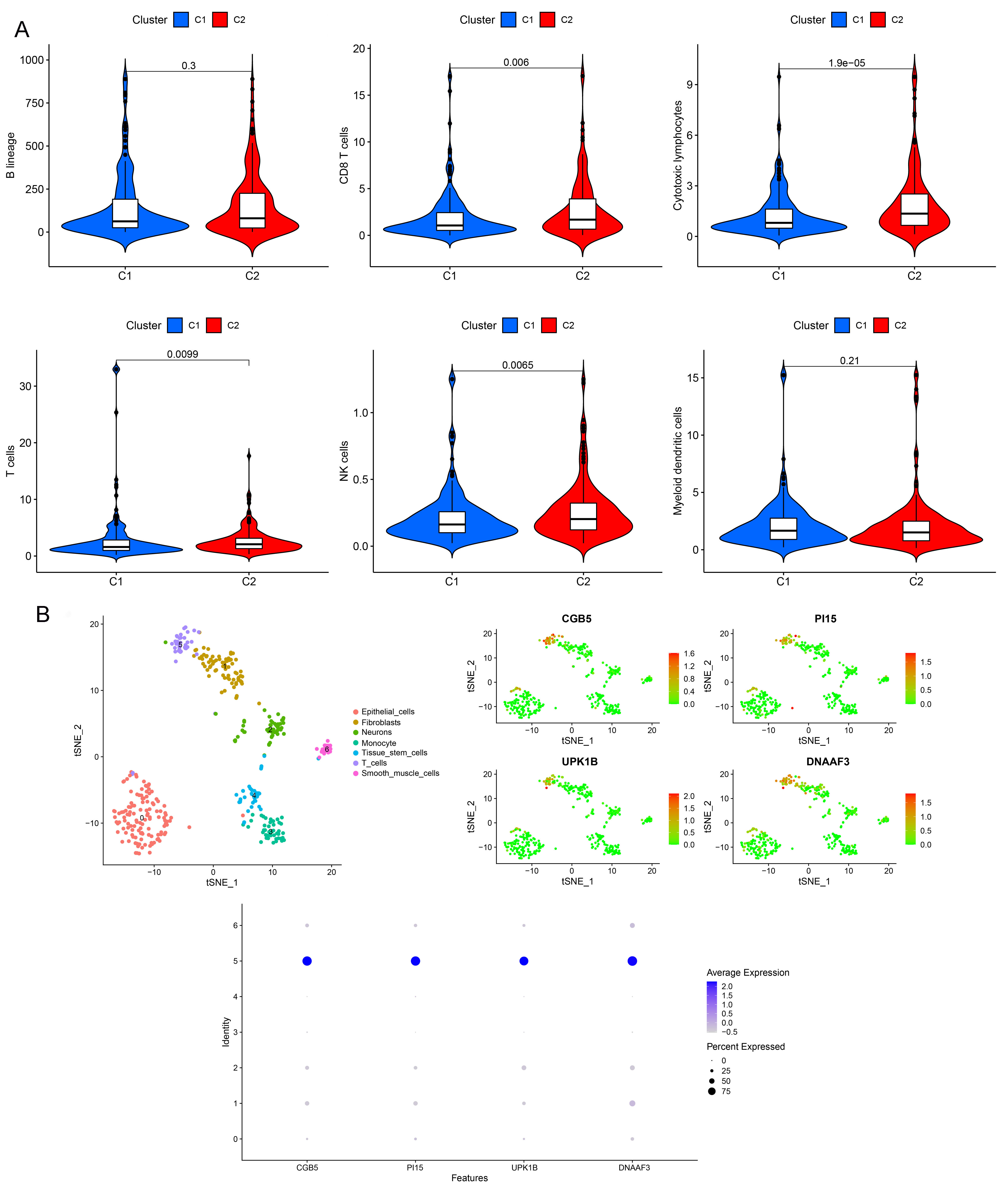

Supplement: Supplementary Figure 1 — (A) Immune cell infiltration between cluster 1 and 2. (B) Expression of signature genes in tumor immune microenvironment by single-cell sequencing analysis. [file Image_1.jpeg]

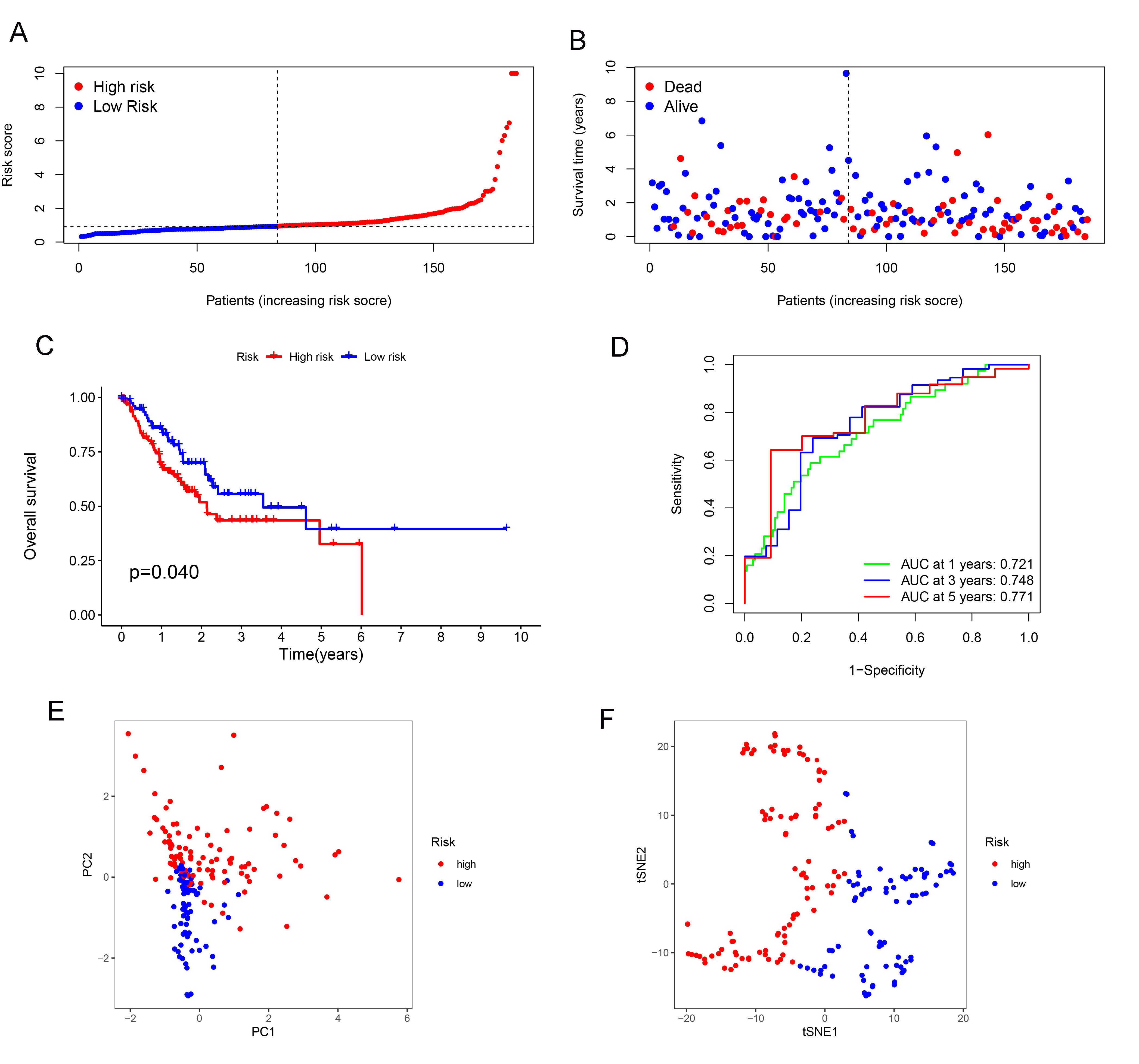

Supplement: Supplementary Figure 2 — Verification of the T cell-positive regulator-related score in TCGA-test. (A) Distribution of risk score in TCGA-test. (B) Survival status plot and survival time of low- and high-risk gastric cancer in TCGA-test. (C) Survival analysis of low- and high-risk gastric cancer in TCGA-test. (D) Survival analysis of low- and high-risk gastric cancer in TCGA-test. (E) PCA analyses of low- and high-risk gastric cancer in TCGA-test. (F) t-SNE analysis of low- and high-risk gastric cancer in TCGA-test. [file Image_2.jpeg]

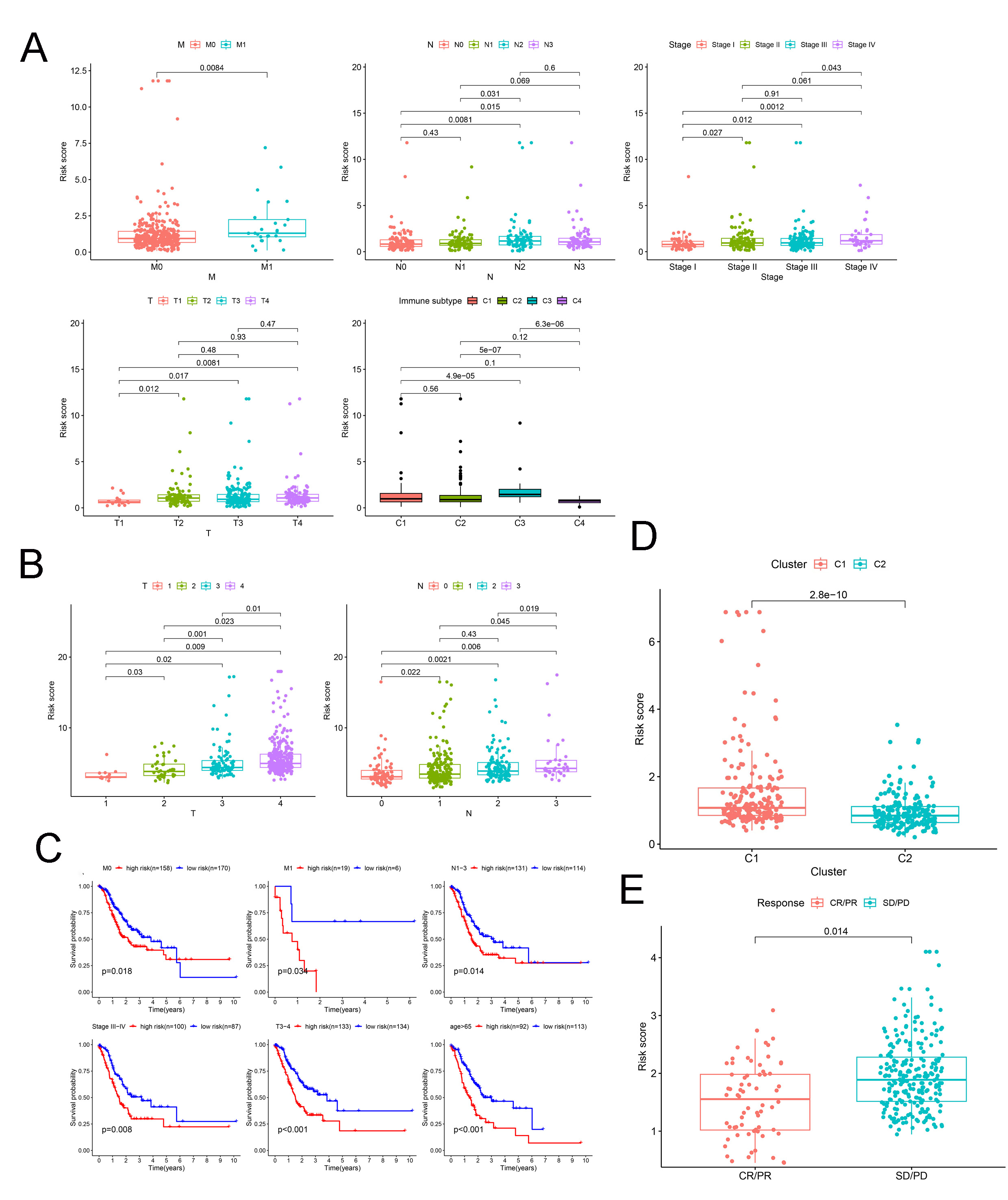

Supplement: Supplementary Figure 3 — (A) Subgroup analysis for the difference of T cell-positive regulator-related risk score in TCGA-STAD dataset. (B) Subgroup analysis for the difference of T cell-positive regulator-related risk score in GEO dataset. (C) Survival analysis of low- and high-risk gastric cancer in different subgroups. (E) Risk score between C1 and C2 clusters. (D) Immunotherapy response prediction across IMvigor210 cohort. [file Image_3.jpeg]
